# Percutaneous Coronary Intervention Without Sustained Return of Spontaneous Circulation Under Extracorporeal Cardiopulmonary Resuscitation: A Phenotype-Oriented Descriptive Systematic Review

**DOI:** 10.3390/jcm15124422

**Published:** 2026-06-07

**Authors:** Yi-Hsiang Chao, Zhi-Hao Tay, Chong-Chao Hsieh

**Affiliations:** 1 Division of Cardiovascular Surgery, Department of Surgery, Kaohsiung Medical University Hospital, Kaohsiung Medical University, Kaohsiung 807, Taiwan; chao.yi.hsiang.md@gmail.com (Y.-H.C.); zhihaotay1911998@gmail.com (Z.-H.T.); 2 Department of Surgery, Faculty of Medicine, College of Medicine, Kaohsiung Medical University, Kaohsiung 807, Taiwan; 3 Graduate Institute of Clinical Medicine, College of Medicine, Kaohsiung Medical University, Kaohsiung 807, Taiwan

**Keywords:** extracorporeal cardiopulmonary resuscitation, ECPR, percutaneous coronary intervention, cardiac arrest, return of spontaneous circulation, VA-ECMO, systematic review

## Abstract

**Background:** Percutaneous coronary intervention (PCI) during extracorporeal cardiopulmonary resuscitation (ECPR) before sustained return of spontaneous circulation (ROSC) is increasingly performed, yet most published reports fail to document ROSC status at the time of intervention—leaving this specific clinical phenotype poorly characterized. We aimed to clarify this ambiguity by systematically separating studies with explicit no-ROSC documentation from those in which the phenotype is only inferred and to describe selection, feasibility, and outcomes for the resulting cohorts. **Methods:** PubMed, Embase, and Cochrane CENTRAL were searched on 30 January 2026. Studies were pre-classified as DEFINITE (explicit no-sustained-ROSC documentation at PCI) or PROBABLE (workflow strongly implying no sustained ROSC). The 13 DEFINITE studies served as the **primary analysis population**; the 14 PROBABLE studies provided supportive evidence. Risk of bias was assessed using ROBINS-I (DEFINITE, primary) and JBI checklists (all studies). Sensitivity analyses excluded overlapping registries (ELSO, SAVE-J). Data were synthesized descriptively along three axes—selection, feasibility, and outcomes—without meta-analysis. Registered in PROSPERO (CRD420251252255); PRISMA 2020 compliant. **Results:** Twenty-seven studies encompassing 12,882 patients were included. In the DEFINITE primary cohort (13 studies, *N* = 3320), median survival to discharge was 30.3% (IQR 26.5–40.8; range 21.0–69.0; and *n* = 11) and favourable neurological outcome (CPC 1–2) 33.5% (IQR 16.8–45.8; range 10.4–92.0; and *n* = 12). Exclude-overlap sensitivity analysis (19 studies, *N* = 2741) yielded concordant estimates (survival 31.1%, IQR 27.2–37.0). PCI rates spanned 24–100% and post-procedural TIMI 3 flow 62.4–84.0%. ROBINS-I rated 9/13 DEFINITE studies at serious overall risk of bias and 4/13 at moderate (none low), predominantly from confounding by indication and selection bias—substantially more stringent than the JBI appraisal. **Conclusions:** PCI without sustained ROSC under ECPR is technically feasible, but the practice is widespread while remaining insufficiently standardized in ROSC reporting. Descriptive benchmarks from DEFINITE studies provide realistic outcome ranges for shared decision-making; no inference regarding comparative effectiveness is possible from observational data. Standardized documentation of ROSC status at PCI initiation is an immediate priority for future ECPR research.

## 1. Introduction

Cardiac arrest remains a leading cause of mortality worldwide, with survival rates for out-of-hospital cardiac arrest (OHCA) typically 5–15% [1]. Because a substantial proportion of patients never achieve sustained return of spontaneous circulation (ROSC) with conventional cardiopulmonary resuscitation (CPR)—a condition termed refractory cardiac arrest—extracorporeal cardiopulmonary resuscitation (ECPR) has emerged as a rescue strategy. ECPR, defined as initiation of venoarterial extracorporeal membrane oxygenation (VA-ECMO) during ongoing cardiac arrest, decouples haemodynamic stabilization from ROSC achievement and is now conditionally recommended in guidelines for selected patients at centres with appropriate infrastructure [2,3,4,5,6].

This decoupling has created a specific clinical phenotype that is the focus of the present review as follows: **percutaneous coronary intervention (PCI) performed while the patient remains ECMO-dependent, without sustained ROSC having been achieved.** At the catheterisation laboratory door—patient cannulated, perfusion entirely machine-dependent, and neurological prognosis uncertain—the team must decide whether to proceed with coronary intervention. Acute coronary syndrome underlies 60–80% of cardiac arrests with a cardiac cause [7], and PCI is standard therapy for acute myocardial infarction; however, PCI protocols were developed in haemodynamically stabilized, ROSC-achieved populations.

**The key methodological problem addressed by this review is that most ECPR reports describe PCI without documenting ROSC status at the time of intervention.** When outcomes are aggregated across cohorts in which some, none, or an unspecified fraction of patients had sustained ROSC at PCI, the true role of revascularisation under ongoing ECMO support becomes impossible to isolate. This reporting gap has been noted in the broader resuscitation literature [8] but has not been addressed for the ECPR-PCI phenotype specifically. The clinical consequence is that clinicians facing this decision lack benchmarks for selection, feasibility, or realistic outcome expectations; the research consequence is that prospective trials cannot build on a characterized baseline population.

To address this gap, we conducted a descriptive systematic review with an explicit methodological clarification as follows: studies were pre-classified as **DEFINITE** (explicit no-ROSC documentation) or **PROBABLE** (workflow-inferred), with the DEFINITE cohort serving as the primary analysis population. Our objectives, organized along three axes, were (1) to describe the selection characteristics of patients who undergo PCI in this scenario (**Axis 1: Selection Profile**); (2) to assess procedural feasibility, including PCI completion rates, reperfusion success, and complications (**Axis 2: Procedural Feasibility**); and (3) to summarize the range of observed clinical outcomes, including survival and neurological recovery (**Axis 3: Outcome Envelope**). This review is explicitly descriptive and exploratory; no meta-analysis was conducted, and no causal inferences regarding the effectiveness of PCI in this population are drawn.

## 2. Methods

### 2.1. Protocol and Registration

This review was registered in PROSPERO (ID: CRD420251252255) and conducted in accordance with PRISMA 2020 guidelines [9]. We deliberately designate this work as a **descriptive systematic review** rather than a scoping review, because the conduct followed the full set of systematic-review methodological standards—pre-registered protocol with explicit inclusion/exclusion criteria, comprehensive multi-database search with documented strategy, dual-reviewer full-text screening (100% of included studies independently re-screened), structured data extraction, and formal risk-of-bias assessment using validated tools (ROBINS-I and JBI; see Section 2.5). What distinguishes the present work from a meta-analytic systematic review is not its methodology but its synthesis: given the heterogeneity in patient populations, ECPR protocols, and outcome definitions across the included studies, descriptive synthesis without pooled estimates is methodologically appropriate, whereas pooled effect estimates would convey false precision. We therefore report descriptive central tendency and ranges across study-level proportions rather than meta-analytic summary effects.

### 2.2. Search Strategy

A systematic search was conducted across the following three electronic databases on 30 January 2026: PubMed/MEDLINE, Embase, and Cochrane Central Register of Controlled Trials (CENTRALs). The search strategy combined three conceptual domains using Boolean operators as follows:

1. **Cardiac arrest:** “cardiac arrest,” “heart arrest,” “cardiopulmonary resuscitation,” “CPR,” “refractory cardiac arrest,” “refractory VF,” “refractory VT”;
2. **ECPR/ECMO:** “ECPR,” “extracorporeal CPR,” “E-CPR,” “ECMO,” “extracorporeal membrane oxygenation,” “VA-ECMO,” “extracorporeal life support,” “ECLS”;
3. **PCI/Angiography:** “percutaneous coronary intervention,” “PCI,” “coronary angiography,” “cardiac catheterization,” “angioplasty,” “coronary revascularization”.

No language or date restrictions were applied at the search stage to maximize sensitivity; however, eligibility required English-language full-text availability. The search was limited to publications from January 2000 onward, as VA-ECMO for cardiac arrest was not widely reported before this date. Full search strategies for each database are provided in Supplementary Materials. Reference lists of all included studies were hand-searched for additional relevant publications. Grey literature sources (conference proceedings, trial registries, and preprint servers) were not systematically searched because the phenotype of interest is not indexed in trial registries, and conference abstracts were excluded by protocol; this is acknowledged as a potential source of publication bias. The restriction to English-language full texts introduces a language bias after an initially unrestricted search; non-English records identified at the search stage are catalogued in the Supplementary Materials but excluded from the primary analysis.

### 2.3. Study Selection

#### 2.3.1. Eligibility Criteria

Studies were eligible for inclusion if they met all of the following criteria: (1) enrolled adults (≥18 years) with out-of-hospital or in-hospital cardiac arrest; (2) used ECPR, defined as VA-ECMO initiated during ongoing cardiac arrest; (3) reported coronary angiography or PCI; (4) included ≥5 patients meeting the phenotype definition; and (5) were published in English. Studies were excluded if they were case reports or case series with fewer than five patients, described VA-ECMO initiated after sustained ROSC (non-ECPR indication), reported only surgical revascularization without catheterization, or did not permit determination of ROSC status at the time of PCI. Non-English studies were catalogued in Supplementary Materials but excluded from the primary analysis.

#### 2.3.2. Phenotype Definition

The target phenotype was defined as patients undergoing PCI at a time when sustained ROSC had not been achieved, with hemodynamic support provided entirely by VA-ECMO. For operational purposes, **sustained ROSC was defined by this review team as ≥20 min of spontaneous circulation without the need for further chest compressions.** We explicitly acknowledge that this ≥20 min cut-off is an **author-defined operational threshold** rather than a formally specified Utstein criterion: the Utstein reporting framework [8] defines sustained ROSC qualitatively and without a fixed numeric duration. The threshold was adopted to provide a reproducible rule for classifying heterogeneous narrative descriptions and is used consistently throughout this review; its limitations are discussed in the Section 4.4.2. Intermittent ROSC episodes were permitted and did not alter classification, provided sustained ROSC had not been established. Electrical activity without mechanical circulation (e.g., pulseless electrical activity, organized rhythm without pulses) was not considered ROSC.

#### 2.3.3. Phenotype Classification

Studies were classified into the following two categories based on ROSC documentation:

- **DEFINITE:** The study explicitly stated that sustained ROSC had not been achieved at the time of coronary intervention, using language such as “no ROSC,” “ongoing CPR at catheterization,” or equivalent documentation.
- **PROBABLE:** The described clinical workflow strongly implied that sustained ROSC had not been achieved (e.g., “cannulated during CPR → immediate transfer to catheterization laboratory”) without contradictory information, but without an explicit statement regarding ROSC status.

Studies in which ROSC status at the time of PCI could not be determined were classified as UNCERTAIN and excluded from the main analysis, with reasons documented in Supplementary Materials. All phenotype classifications were recorded with supporting textual evidence (direct quotations, page numbers, and table or figure references) in a structured decision log.

#### 2.3.4. Screening Process

Title and abstract screening was performed using a three-element inclusion criterion requiring the concurrent presence of cardiac arrest, ECPR/ECMO, and coronary angiography/PCI. Screening was conducted by one reviewer (Y.H.C.) with structured assistance from a large language model (Claude, Anthropic PBC). The AI tool functioned as a **second-pass recommendation engine, not an adjudicator**: for each record, the AI was supplied with the pre-specified inclusion and exclusion criteria and produced (a) a preliminary include/exclude suggestion, (b) the triggering inclusion-criterion element(s), and (c) a one-sentence rationale. The human reviewer (Y.H.C.) independently evaluated every record against the criteria—not only records flagged by the AI—and made all final screening decisions, which overrode any AI suggestion. A second reviewer (Z.H.T.) independently re-screened a random 10% stratified sample of title/abstract records (stratified by AI-suggested decision) to audit concordance; disagreements were resolved by discussion. This AI-assisted workflow is intended to improve **sensitivity** during the high-volume title/abstract stage; it does not substitute for dual independent human screening at the full-text stage.

Full-text screening was performed by the primary reviewer (Y.H.C.) without AI assistance. A second reviewer (Z.H.T.) independently re-screened **100% of studies classified as included** and a random 20% sample of excluded studies. Disagreements were resolved through discussion until consensus was reached.

For phenotype classification, the primary reviewer performed initial classification with AI-assisted drafting that produced structured outputs including direct textual quotations from the full text, page or figure references, a DEFINITE/PROBABLE/UNCERTAIN label, and a stated confidence level. All classifications, together with the supporting evidence, were subsequently reviewed and confirmed by Y.H.C. based on the source text; the AI classifications were used as structured drafts, not as final judgements. We did not calculate a formal inter-rater reliability (κ) statistic for classification because the verification workflow was sequential rather than fully independent and κ values from non-independent adjudication are known to be misleadingly inflated. Instead, **we adopted the following pre-specified DEFINITE-primary analytic strategy: the 13 DEFINITE studies (explicit ROSC documentation) constitute the primary analysis cohort, while the 14 PROBABLE studies (workflow-inferred phenotype) are reported as a supportive cohort.** The consistency of findings between the two cohorts, and between the combined set and the exclude-overlap subset, is examined in sensitivity analyses (see Section 2.6).

### 2.4. Data Extraction

Data were extracted by one reviewer (Y.H.C.) using a structured extraction form covering three domains aligned with the study’s three-axis framework. Domain 1 (Selection Profile) included demographics (age, sex), arrest characteristics (setting, witnessed status, bystander CPR, and initial rhythm), time intervals (low-flow time with definition specification), and ACS suspicion indicators. Domain 2 (Procedural Feasibility) included angiography and PCI rates, culprit lesion identification, reperfusion success (TIMI flow grade), adjunctive mechanical support, and procedural complications. Domain 3 (Outcome Envelope) included survival to hospital discharge, neurological outcome (Cerebral Performance Category 1–2), ECMO weaning and duration, and post-PCI ROSC.

For studies with overlapping populations, data were extracted from complementary axes to minimize double-counting while maximizing information yield. A second reviewer (Z.H.T.) verified extraction accuracy for a random 20% sample of included studies, with discrepancies resolved through discussion. All assumptions and inferences made during extraction were documented in a separate assumptions log with supporting evidence and reasoning.

### 2.5. Quality Assessment

Risk of bias was assessed with two tools applied in a complementary fashion, reflecting the heterogeneous designs of the included studies and in response to peer-review concerns that a reporting-quality checklist alone may overstate the methodological robustness of retrospective observational data.

**Primary risk-of-bias tool—ROBINS-I.** The Risk Of Bias In Non-randomized Studies of Interventions (ROBINS-I) tool [10] was applied to the **13 DEFINITE studies** (primary analysis cohort) across the following seven domains: (1) confounding, (2) selection of participants, (3) classification of interventions, (4) deviations from intended interventions, (5) missing data, (6) measurement of outcomes, and (7) selection of the reported result. Each domain was judged *Low*, *Moderate*, *Serious*, *Critical*, or *No Information*; the overall judgement equals the worst single-domain rating per the ROBINS-I algorithm. ROBINS-I is specifically designed for non-randomized studies of interventions and explicitly probes confounding by indication, selection bias, and immortal-time considerations, which are the dominant methodological risks in observational studies of an acutely selected intervention like PCI during ongoing ECPR.

**Supplementary risk-of-bias tool—JBI Critical Appraisal Checklists.** The JBI checklists [11] were applied to **all 27 included studies** and are reported in the Supplementary Materials. A dual-tool strategy was employed as follows: the JBI Checklist for Case Series (10 items) was applied to single-arm descriptive studies, and the JBI Checklist for Cohort Studies (2024 revision, 11 items) was applied to studies with comparison groups. Each item was scored as “Yes,” “No,” “Unclear,” or “Not Applicable”; overall risk was categorized as low (≥75% of applicable items scored “Yes”), moderate (50–74%), or high (<50%). The JBI assessment characterizes *reporting quality* and is retained for transparency and cross-study comparability with prior descriptive reviews; it is not used as the primary risk-of-bias statement in this revision.

The ROBINS-I assessment was performed by one reviewer (Y.H.C.) within the constraint of the revision timeline; single-reviewer ROBINS-I assessment is acknowledged as a limitation in Section 4. To partially mitigate this, every domain-level judgement was accompanied by a written 1–2-sentence rationale citing the specific study features that drove the rating (Supplementary Table S7), so that the reasoning is fully transparent and any subsequent reviewer can independently verify or challenge each judgement against the source text. Quality scores from either tool were reported descriptively and were not used as exclusion criteria.

### 2.6. Data Synthesis

Given heterogeneity in study populations, designs, and outcome definitions, data were synthesized descriptively without pooled estimates. Categorical variables are presented as counts and percentages; continuous variables as medians with interquartile ranges or means with standard deviations as reported in the original studies. Results are organized by the three-axis framework (Selection Profile, Procedural Feasibility, and Outcome Envelope) and presented in summary tables with narrative synthesis. All quantitative displays are labelled as exploratory or descriptive. **All central tendency estimates are unweighted medians across study-level proportions**, not patient-weighted pooled proportions; this preserves each study’s equal status as a descriptive observation and avoids giving disproportionate influence to the largest registries.

**Primary cohort.** The 13 DEFINITE studies served as the primary analysis population, with central tendency (median and IQR) and range reported for all key outcomes.

**Supportive cohort.** The 14 PROBABLE studies were analyzed in parallel as a supportive cohort; estimates from the combined 27-study set are reported alongside for transparency. The combined set should be interpreted in light of the phenotype classification uncertainty in PROBABLE studies.

**Pre-specified sensitivity analyses.** The following two sensitivity analyses were performed to assess the robustness of the descriptive envelope:

1. **DEFINITE-only vs. combined.** Key outcomes (survival to discharge, favourable neurological outcome, PCI rate, and TIMI 3 flow) were compared between the DEFINITE primary cohort and the combined 27-study set to quantify the influence of phenotype-classification uncertainty.
2. **Exclude-overlap.** To address the acknowledged inter-registry overlap (ELSO, SAVE-J II and their secondary analyses; maximum overlap ≈ 1094 patients, 8.5% of the combined N), we repeated the key-outcome synthesis after excluding studies flagged as having overlapping patient populations. This yields an exclude-overlap subset (*n* = 19 studies, *N* = 2741 patients) in which double counting is avoided.

For both sensitivity analyses, consistency with the primary DEFINITE cohort was interpreted as evidence that the descriptive findings are not driven by phenotype-classification uncertainty or by double-counted registry patients.

### 2.7. Deviations from Protocol

Three deviations from the registered protocol occurred during the conduct of this review. First, the quality assessment tool was initially changed from the Newcastle–Ottawa Scale to JBI Critical Appraisal Checklists, as the predominantly single-arm study designs were better suited to a descriptive appraisal framework than one designed for comparative cohorts. Second, during peer-review revision, **ROBINS-I was added as the primary risk-of-bias tool for the 13 DEFINITE studies** in response to reviewer concerns that a reporting-quality checklist alone may understate the inherent methodological risks of retrospective observational data on an acutely selected intervention; the JBI assessment was retained for all 27 studies in the Supplementary Materials. Third, formal inter-rater reliability testing for phenotype classification was replaced with transparent process reporting and the DEFINITE-primary/PROBABLE-supportive framework plus exclude-overlap sensitivity analysis, as the sequential verification workflow did not satisfy the independence assumption required for meaningful kappa calculation.

## 3. Results

### 3.1. Study Selection Results

The systematic search across PubMed, Embase, and Cochrane CENTRAL identified 2741 records (PubMed: 1120; Embase: 1602; and Cochrane: 19). After removing 346 duplicates, 2395 unique records underwent title and abstract screening using a three-element inclusion criterion requiring the concurrent presence of cardiac arrest, ECPR, and coronary angiography/PCI. Of these, 122 records met criteria for full-text review. Three records were excluded as conference abstracts prior to full-text retrieval, and seven full-text articles were unavailable despite institutional access attempts. Full-text screening of 112 articles yielded 31 potentially eligible studies.

**Figure 1:**
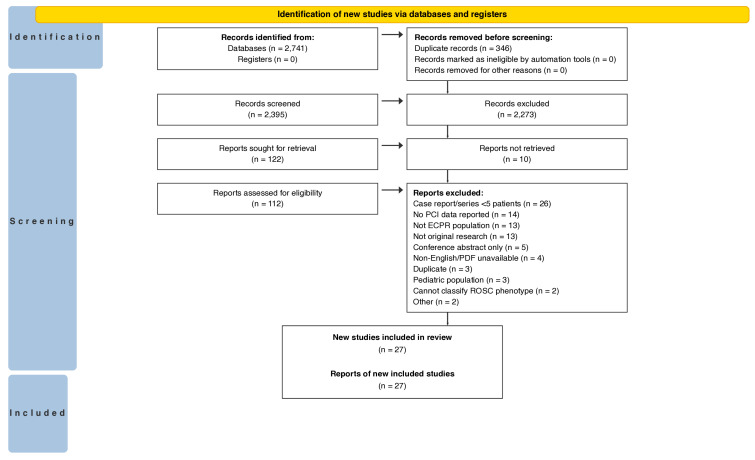
PRISMA 2020 flow diagram showing study identification and selection. Records identified: 2741 (PubMed 1120, Embase 1602, Cochrane CENTRAL 19). After removing 346 duplicates, 2395 records were screened. Full-text screening of 112 articles yielded 27 included studies (13 DEFINITE, 14 PROBABLE).

During phenotype classification, the following four additional studies were excluded: one described post-cardiac arrest shock rather than refractory arrest, one was a single-patient case report, and two lacked any coronary intervention data despite being ECPR cohorts. The final sample comprised 27 studies (Figure 1).

### 3.2. Study Characteristics

**Figure 2:**
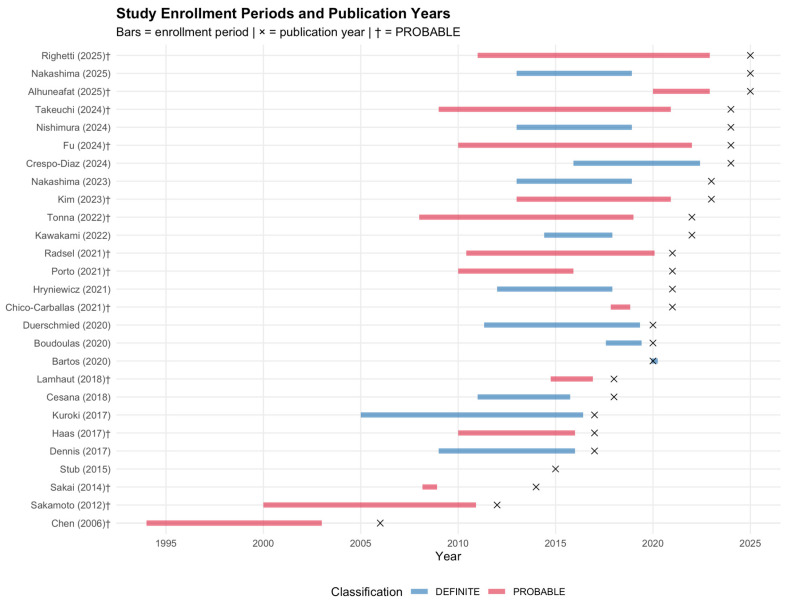
Study enrollment periods (horizontal bars) and publication years (×). Colour indicates phenotype classification (blue = DEFINITE, red = PROBABLE). Studies shown: refs [12,13,14,15,16,17,18,19,20,21,22,23,24,25,26,27,28,29,30,31,32,33,34,35,36,37,38].

The 27 included studies were published between 2006 and 2025, encompassing 10 countries across four continents (Table 1). Japan contributed the largest number of studies (*n* = 8), followed by the United States (*n* = 4) and Italy (*n* = 3). Three additional studies were international multicenter registries. Study designs included retrospective cohorts (*n* = 13), registries (*n* = 10, including one secondary analysis), prospective cohorts (*n* = 3), and a case series (*n* = 1). Most were from single centres (*n* = 14), with the remainder being multicenter or registry-based. The aggregate phenotype population comprised 12,882 patients, with individual study sample sizes ranging from 7 to 7488. Enrollment periods spanned from 1994 to 2022, with the majority commencing after 2010 (Figure 2).

#### 3.2.1. Phenotype Classification and Analytic Cohorts

Of the 27 included studies, **13 were classified as DEFINITE** (explicit documentation of no sustained ROSC at PCI initiation; aggregate *N* = 3320) and **14 as PROBABLE** (clinical workflow strongly implying no sustained ROSC; aggregate *N* = 9562). In this revised analysis, **the 13 DEFINITE studies constitute the primary analysis cohort**; the 14 PROBABLE studies are reported as a supportive cohort, and combined estimates across all 27 studies are shown alongside for transparency.

#### 3.2.2. Methodological Caveats (Read Before Numeric Sections)

The following three interpretative caveats apply to all descriptive estimates that follow and are made prominent here rather than later in the results narrative:

1. **Phenotype-classification uncertainty (PROBABLE cohort).** In 14 of 27 studies, ROSC status at PCI was inferred from described clinical workflow rather than explicitly stated; consequently, combined 27-study estimates carry classification uncertainty that the DEFINITE primary cohort does not.
2. **Overlapping populations across registries.** Three groups of overlapping populations were identified. Two studies from the SAVE-J II registry [14,15] and one subsequent analysis [12] shared the same source population (maximum overlap ≈ 877 patients); two ELSO registry studies [30,35] had temporally overlapping enrolment periods (maximum overlap ≈ 217 patients), while a third ELSO study [25] did not overlap temporally; and three studies included mixed cardiac arrest and cardiogenic shock cohorts with only partial subgroup data. The aggregate combined N of 12,882 may therefore include up to approximately **1094 patients (~8.5%) counted more than once** across overlapping registries. A pre-specified exclude-overlap sensitivity analysis (Section 3.5.5) assesses the impact.
3. **Heterogeneous endpoint definitions.** Survival definitions varied (hospital discharge, ICU discharge, and 30-day) and favourable neurological outcome was assessed at different timepoints across studies; all numeric ranges below should be interpreted with this heterogeneity in mind.

### 3.3. Axis 1: Selection Profile

#### 3.3.1. Demographics

Across all studies, the median or mean age ranged from 52 to 72 years, and the proportion of male patients ranged from 57.1% to 93.8% (25 studies reporting; Table 1).

#### 3.3.2. Arrest Setting

The majority of studies enrolled OHCA patients. Among 23 studies reporting arrest setting, OHCA proportions ranged from 0% (one IHCA-only study) to 100% (14 pure OHCA studies), with eight studies enrolling mixed OHCA/IHCA populations. Witnessed arrest was reported in 12 studies, with proportions ranging from 72.3% to 100%; the high rates in some cohorts reflected ECPR eligibility criteria requiring witnessed arrest. Bystander CPR was reported in nine studies, ranging from 37.1% to 100%.

#### 3.3.3. Initial Cardiac Rhythm

Initial shockable rhythm (VF/pVT) was reported in 22 studies, ranging from 23.5% to 100%. Five studies enrolled exclusively shockable-rhythm patients by protocol. Overall, the majority of studies reported shockable rates exceeding 70%, consistent with typical ECPR selection criteria.

#### 3.3.4. Low-Flow Time

Low-flow time was reported in 20 studies, with values ranging from 25.5 to 78.0 min. The definition of low-flow time varied substantially as follows: eight studies used collapse-to-ECMO initiation, four used call-to-ECMO time, three used CPR duration, and five used other or unspecified definitions. This heterogeneity in definitions limits direct comparisons across studies (Table 1 footnotes).

#### 3.3.5. ACS Suspicion

Suspected acute coronary syndrome was reported in 15 studies (range: 30.0–100%). STEMI rates were reported in seven studies (range: 34.1–92.0%). Several studies enrolled exclusively ACS populations by design, precluding the ACS rate from serving as a meaningful selection variable in those cohorts.

### 3.4. Axis 2: Procedural Feasibility

#### 3.4.1. PCI Rates

**Figure 3:**
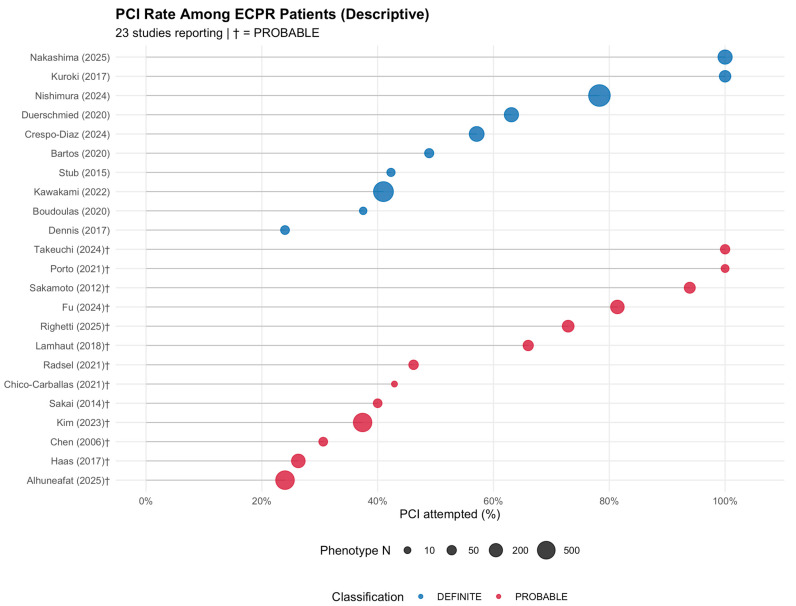
Descriptive PCI rates among ECPR patients across reporting studies. Studies shown: refs [12,13,14,15,16,17,18,19,20,21,22,23,24,25,26,27,28,29,30,31,32,33,34,35,36,37,38].

Among 23 studies reporting PCI utilization data, PCI rates ranged from 24.0% to 100% of the phenotype population (Table 2; Figure 3). In seven studies, all patients underwent PCI by design, reflecting protocols in which coronary intervention was integral to the ECPR workflow. In registry-based studies, PCI rates were generally lower—24.0% in one ELSO analysis [25] and 26.3% in another [35]—reflecting broader, unselected populations and substantial regional variation (Europe 35.6% vs. North America 9.4%) [35].

#### 3.4.2. Reperfusion Success

Post-procedural TIMI 3 flow was reported in five studies, ranging from 62.4% to 84.0%. Reperfusion success (TIMI ≥ 2) was reported in two additional studies at 84.0% and 87.0%, respectively. Kuroki et al. [23] observed a graded association between time to reperfusion and 30-day neurological outcome, with favourable outcomes in 74% of patients reperfused within 60 min versus 23% when reperfusion exceeded 90 min.

#### 3.4.3. Culprit Lesion Distribution

Culprit lesion identification was reported in 10 studies (range: 53.3–100%). The left anterior descending artery was the most frequently involved vessel (27–53%), followed by the right coronary artery (17–25%) and left main artery (4–34%). The high left main involvement in some studies (e.g., 34% in [23]) underscores the severity of coronary disease in this population.

#### 3.4.4. Time to Coronary Intervention

Time from ECMO initiation to coronary intervention was infrequently reported. Fu et al. [27] reported a median ECMO-to-balloon time of 90 min, while Kuroki et al. [23] reported a mean collapse-to-balloon time of 103.2 min. The paucity of data on this variable precludes further characterization.

#### 3.4.5. Adjunctive Mechanical Support

IABP use was reported in 13 studies (range: 5.0–100%), with higher utilization observed in Japanese studies (e.g., 89.6–91.0%). Impella use was infrequently reported and rarely utilized.

#### 3.4.6. Procedural Complications

Complication data were available in 11 studies. Major bleeding was reported in eight studies (range: 1–68 events). Limb ischemia was reported in nine studies (range: 0–24 events), and stroke in seven studies (range: 0–6 events). Importantly, complications in most studies were reported for the entire ECMO cohort rather than the PCI subgroup specifically, limiting the attribution of complications to the coronary intervention itself.

### 3.5. Axis 3: Outcome Envelope (Exploratory)

#### 3.5.1. Survival

**Figure 4:**
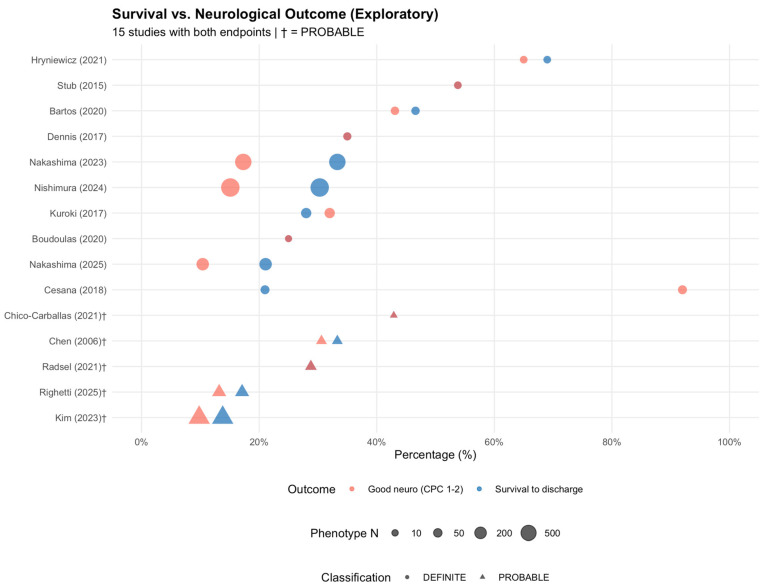
Exploratory range of survival to hospital discharge across reporting studies. Point size proportional to sample size. Colour indicates classification. Studies shown: reporting studies among refs [12,13,14,15,16,17,18,19,20,21,22,23,24,25,26,27,28,29,30,31,32,33,34,35,36,37,38].

**DEFINITE primary cohort (11 studies with survival data, *N* = 3320):** Unweighted median survival to hospital discharge was **30.3% (IQR 26.5–40.8; range 21.0–69.0) (Figure 4)**. Higher rates were observed in highly selected cohorts—for example, 69.0% in a single-centre cohort [17] (which also had 73% in-hospital cardiac arrest, a pronounced selection effect discussed below), 53.8% in a prospective pilot trial [24], and 46.6% in a community-wide VF/VT programme [18]. Larger registry studies generally reported survival in the 20–33% range.

**Supportive (combined 27-study) estimate:** Median 29.2% (IQR 23.1–34.2), range 13.8–69.0 across 20 reporting studies. One additional study reported survival to ICU discharge (17.1%).

#### 3.5.2. Favourable Neurological Outcome

**Figure 5:**
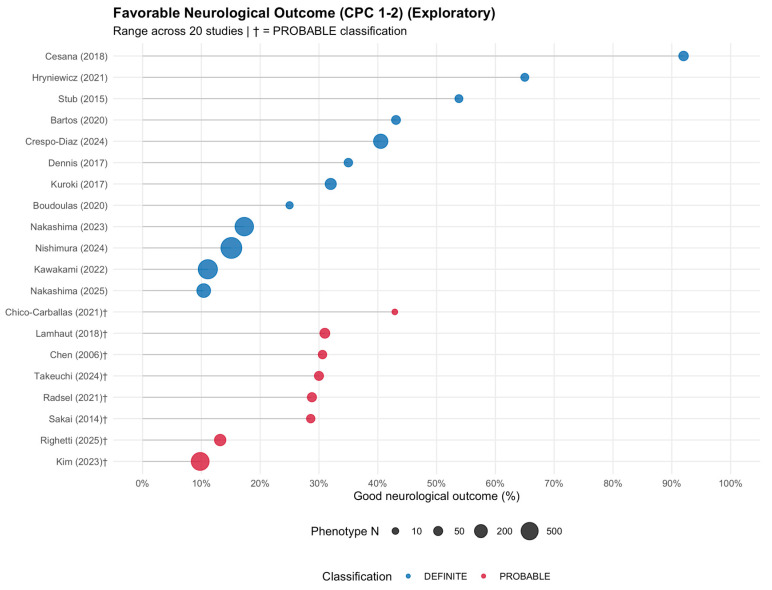
Exploratory range of favourable neurological outcome (CPC 1–2) across reporting studies. Point size proportional to sample size. Studies shown: reporting studies among refs [12,13,14,15,16,17,18,19,20,21,22,23,24,25,26,27,28,29,30,31,32,33,34,35,36,37,38].

**DEFINITE primary cohort (12 studies reporting, *N* = 3320):** Unweighted median favourable neurological outcome (CPC 1–2) was **33.5% (IQR 16.8–45.8; range 10.4–92.0) (Figure 5)**. The highest rate (92.0%) [21] reflected that nearly all survivors in that cohort (12/13) had favourable outcomes, though overall survival was only 21.0% (13/63). In the larger DEFINITE cohorts, favourable neurological outcome fell within the 10–43% range.

**Supportive (combined 27-study) estimate:** Median 30.3% (IQR 16.8–41.1), range 9.8–92.0 across 20 reporting studies. Assessment timing varied as follows: 12 studies at hospital discharge, four at 30 days, two at ICU discharge, and two at other timepoints—this endpoint heterogeneity limits direct comparability and should be read alongside each study’s individual timepoint (Table 3).

#### 3.5.3. ECMO Weaning and Duration

ECMO weaning was reported in nine studies (range: 27.1–69.4%). ECMO duration was reported in 15 studies, with medians or means ranging from approximately 1 to 6 days.

#### 3.5.4. Post-PCI ROSC

Only one study [12] explicitly reported post-PCI ROSC: 163/251 (64.9%) patients achieved sustained ROSC following coronary reperfusion. This variable was not systematically captured in other studies, precluding broader characterization.

#### 3.5.5. Sensitivity Analyses

The following two pre-specified sensitivity analyses were performed (Supplementary Materials, Sensitivity Analysis Table):

1. **DEFINITE vs. combined.** Central tendency and range for survival and favourable neurological outcome were concordant between the DEFINITE primary cohort and the combined 27-study set: DEFINITE median survival 30.3% (IQR 26.5–40.8) versus combined 29.2% (IQR 23.1–34.2); DEFINITE median favourable neurological outcome 33.5% (IQR 16.8–45.8) versus combined 30.3% (IQR 16.8–41.1). The inclusion of PROBABLE studies did not materially shift the observed outcome envelope.
2. **Exclude-overlap subset (19 studies, *N* = 2741).** After excluding studies flagged as having overlapping patient populations (ELSO and SAVE-J II-related studies), key outcomes remained concordant with the DEFINITE primary cohort: median survival 31.1% (IQR 27.2–37.0; range 13.8–69.0; *n* = 12) and median favourable neurological outcome 30.6% (IQR 26.8–37.8; range 9.8–65.0; *n* = 15). This indicates that the descriptive envelope is not an artefact of patient double-counting across registries.

Collectively, the two sensitivity analyses support that the descriptive outcome ranges reported above are not driven by either phenotype-classification uncertainty (PROBABLE inclusion) or by inter-registry overlap.

### 3.6. Risk of Bias Assessment

#### 3.6.1. ROBINS-I—Primary Assessment (13 DEFINITE Studies)

**Figure 6:**
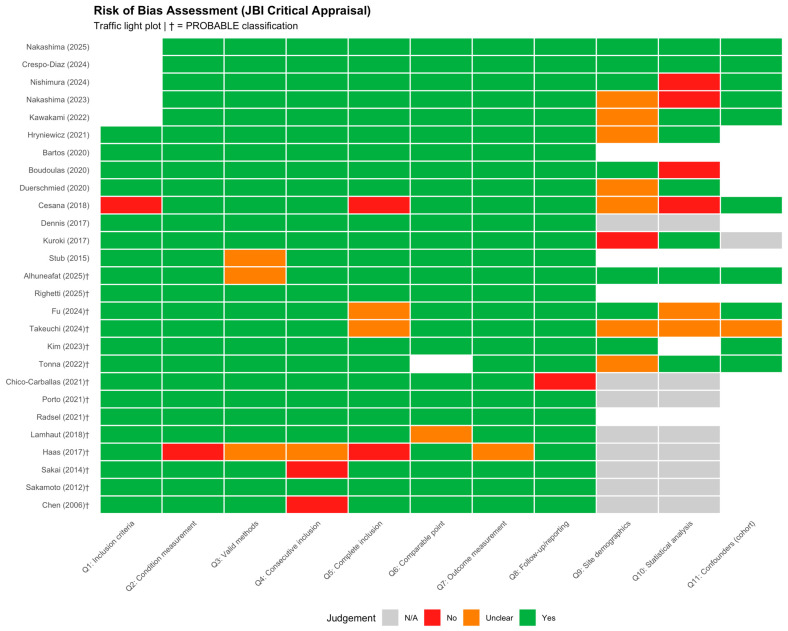
ROBINS-I (Risk Of Bias In Non-randomized Studies of Interventions) traffic-light plot for the 13 DEFINITE studies (primary analysis cohort). Domains: D1—Confounding; D2—Selection of participants; D3—Classification of interventions; D4—Deviations from intended interventions; D5—Missing data; D6—Outcome measurement; D7—Selection of reported results. Overall judgement reflects the worst single-domain rating per the ROBINS-I algorithm. Distribution: 0—Low, 4—Moderate, 9—Serious, 0—Critical. Full domain-level rationale in Supplementary Table S7. The JBI Critical Appraisal results for all 27 studies are summarized in the Supplementary Materials (Table S2 and Figure S1) as a reporting-quality reference. Studies shown: the 13 DEFINITE studies [12,13,14,15,16,17,18,19,20,21,22,23,24].

The ROBINS-I tool was applied to the 13 DEFINITE studies across its seven domains. **Nine studies (69.2%) were rated at overall serious risk of bias and four (30.8%) at overall moderate risk; no study was rated low or critical** (Figure 6; full domain-level judgements in Supplementary Materials). The dominant drivers of serious ratings were (i) **D2—Selection of participants**, including physician-discretion ECPR/PCI activation, mid-study protocol changes that shifted inclusion criteria, and tight selection of favourable-prognosis patients; and (ii) **D1—Confounding** by indication, which is intrinsic to observational studies of an acutely selected intervention such as PCI during ongoing ECPR. Missing data (D5), particularly unexplained denominator shifts and incomplete neurological outcome ascertainment, contributed in several studies. Outcome measurement (D6) was generally well-handled, reaching serious in only one study that did not report any neurological outcome despite being a cardiac arrest cohort. The four moderate-rated studies (Nakashima 2025 [12], Crespo-Diaz 2024 [13], Kawakami 2022 [16], Kuroki 2017 [23]) were larger multicentre or well-protocolized single-centre cohorts with explicit sustained-ROSC definitions, standardized outcome measurement, and multivariable or mixed-effect handling of confounding. No DEFINITE study reached an overall low rating, which reflects the methodological ceiling of retrospective observational data on this acutely selected population rather than deficient individual study conduct.

#### 3.6.2. JBI—Supplementary Assessment (All 27 Studies)

For transparency and comparability with prior descriptive reviews, the JBI Critical Appraisal Checklists were applied to all 27 included studies (Tables S1–S7 in Supplementary). Under the JBI reporting-quality criteria, 24 studies (88.9%) met the ≥75% threshold (low concern), three (11.1%) fell into 50–74% (moderate concern), and none below 50%. The most commonly flagged JBI items were incomplete reporting of arrest circumstances, site-level demographic detail, and unclear consecutive-enrolment verification in registry studies.

The contrast between the two assessments is instructive as follows: JBI captures whether a study reports its methodology transparently, while ROBINS-I probes confounding, selection, and other bias mechanisms that are structural features of this clinical scenario. Reviewers should weight the ROBINS-I assessment—not the JBI—when interpreting the methodological strength of the primary descriptive findings.

## 4. Discussion

### 4.1. Principal Findings

This descriptive systematic review characterized a specific clinical phenotype—PCI performed while the patient remained ECMO-dependent without sustained ROSC—by pre-classifying 27 studies (12,882 patients) into a **DEFINITE primary cohort** (13 studies, *N* = 3320, with explicit no-ROSC documentation) and a **PROBABLE supportive cohort** (14 studies, *N* = 9562, workflow-inferred). This classification strategy directly addresses the methodological gap identified in the Introduction as follows: most published ECPR reports describe PCI without documenting ROSC status at the time of intervention. Three principal findings emerged.

First, the selection profile in the DEFINITE cohort was broadly consistent with typical ECPR eligibility as follows: predominantly male (57–94%), middle-aged to older adults (median/mean age 52–72 years), with out-of-hospital cardiac arrest, initial shockable rhythm, and low-flow times generally 25–78 min. Notable variability in ACS suspicion rates and inclusion stringency across centres reflects different institutional selection philosophies rather than a unified practice pattern.

Second, PCI was technically feasible across the DEFINITE cohort and the wider evidence base: PCI rates ranged from 24% to 100% of the phenotype population, and post-procedural TIMI 3 flow was achieved in 62.4–84.0% of the studies that reported it. Coronary reperfusion is therefore attainable under ECMO support without sustained ROSC.

Third, in the DEFINITE primary cohort the outcome envelope—unweighted median survival to discharge 30.3% (IQR 26.5–40.8; range 21.0–69.0) and favourable neurological outcome 33.5% (IQR 16.8–45.8; range 10.4–92.0)—was concordant with the combined 27-study set and with the exclude-overlap sensitivity subset, supporting the robustness of the descriptive envelope. The width of the ranges reflects substantial heterogeneity in patient selection, institutional protocols, and endpoint definitions and is interpreted here as a feasibility-and-context finding, not evidence of treatment effect.

### 4.2. Interpretation in Context

#### 4.2.1. The Phenotype Is Recognized but Inconsistently Documented

This review confirms that PCI without sustained ROSC under ECPR is a recognized clinical practice described in the published literature. However, the finding that only 13 of 27 studies (48%) explicitly documented ROSC status at the time of coronary intervention highlights a critical gap in reporting standards. The remaining 14 studies required inference from described clinical workflows—a process that, while methodologically transparent, introduces classification uncertainty. This inconsistency is not unique to the ECPR-PCI literature; ROSC documentation has been identified as a broader challenge in resuscitation research, where the dynamic nature of cardiac arrest makes precise temporal documentation difficult [8].

#### 4.2.2. Selection Logic Varies Across Centres

The selection characteristics observed in this review reveal both consistencies and notable variation. The predominance of shockable rhythms (exceeding 70% in most studies) and the requirement for witnessed arrest in many protocols reflect the general ECPR selection paradigm. However, the wide range of low-flow times (25.5–78.0 min) and the heterogeneity in low-flow time definitions (collapse-to-ECMO, call-to-ECMO, CPR duration, and others) complicate comparisons across studies. This definitional inconsistency has been recognized as a major challenge in ECPR research more broadly [8].

The proportion of patients with suspected ACS varied from 34% to 100% across reporting studies, reflecting fundamentally different selection philosophies as follows: some centres perform coronary angiography selectively based on ACS suspicion, while others adopt a routine early angiography approach for all ECPR patients regardless of suspected etiology. This variation mirrors the ongoing debate in the broader post-cardiac arrest literature regarding the role of immediate versus selective coronary angiography [39] and is further complicated by recent registry evidence that STEMI and non-STEMI presentations, while initially distinct in reinfarction rates, converge in one-year outcomes after statistical adjustment [40]—suggesting that prognostic differences relevant to ECPR selection cannot be inferred from the electrocardiographic phenotype alone.

#### 4.2.3. Feasibility Is Supported but Context-Dependent

The observation that PCI can be completed under ECMO support without sustained ROSC is an important, if expected, finding ECMO provides sufficient hemodynamic stability to permit fluoroscopy-guided coronary intervention, and the TIMI 3 flow rates observed (62–84%, from five reporting studies) approach, though fall below, the >90% rates reported in conventional primary PCI for ST-elevation myocardial infarction. However, PCI rates varied substantially, from 24% in large registry studies to 100% in protocol-driven single-centre cohorts. The lower rates in registry-based studies likely reflect the heterogeneity of real-world practice, where not all ECPR patients undergo coronary angiography and not all angiograms lead to intervention.

The regional variation observed—for example, PCI rates of 35.6% in Europe versus 9.4% in North America in one international registry [35]—suggests that institutional and regional practice patterns, rather than patient-level factors alone, drive the decision to pursue coronary intervention under these circumstances. Japanese studies reported notably high rates of intra-aortic balloon pump (IABP) use (89–91%), reflecting a practice pattern that persists in Japan despite contemporary western trends away from routine IABP use following the IABP-SHOCK II trial [41] (which demonstrated no benefit in cardiogenic shock, though notably not in the cardiac arrest population studied here).

Complication data, while limited, identified major bleeding as the most commonly reported adverse event. However, most studies reported complications for the entire ECMO cohort rather than the PCI subgroup specifically, precluding definitive attribution of complications to the coronary intervention itself versus the ECMO cannulation, anticoagulation, or other concurrent procedures. Notably, two clinically important variables—anticoagulation management during ECMO-supported PCI (balancing circuit thrombosis against procedural bleeding) and coronary access site strategy (given that femoral access may be occupied by ECMO cannulae)—were not systematically reported in any included study, representing important gaps for future investigation.

#### 4.2.4. Outcome Ranges Are Wide and Context-Dependent

The survival and neurological outcome ranges observed in this review span a wide spectrum that defies simple summarization. The highest survival rates (53–69%) were observed in highly selected, single-centre cohorts with protocol-driven approaches—for example, Hryniewicz et al. [17] reported 69% survival in a cohort of only 26 patients with predominantly in-hospital cardiac arrest (73% IHCA), reflecting severe selection bias—while larger registry studies generally reported rates of 20–33%. This pattern is consistent with the well-described centre effect in ECPR outcomes, where experience, patient selection criteria, and post-resuscitation care protocols substantially influence results [30].

The finding that favourable neurological outcome rates closely tracked survival rates in most studies—with the majority of survivors achieving CPC 1–2—suggests that neurological recovery is possible in this population, although the direction of causality cannot be inferred. It may be that patients who survive are those with less severe neurological injury at baseline, rather than PCI conferring neurological protection.

The two pre-specified sensitivity analyses—DEFINITE-primary versus combined-27 and exclude-overlap (*n* = 19 studies, *N* = 2741)—produced concordant estimates (Section 3.5). Median survival and favourable neurological outcome in each subset fell within overlapping IQRs, supporting that the descriptive envelope is not an artefact of phenotype-classification uncertainty from PROBABLE studies or of patient double-counting across ELSO and SAVE-J II-related datasets. This consistency should not be over-interpreted: it quantifies the absence of a large overlap-driven or misclassification-driven effect on the observed envelope, not the absence of residual selection or confounding bias captured separately by the ROBINS-I assessment.

### 4.3. Clinical Implications (Exploratory)

The following implications are exploratory and hypothesis-generating. No causal inference is possible from descriptive observational data.

#### 4.3.1. Informing the Decision at the Catheterization Laboratory Door

For clinicians facing the decision of whether to proceed with PCI in an ECMO-dependent patient without sustained ROSC, this review provides descriptive context rather than prescriptive guidance. The data suggest that the procedure is technically feasible across a range of clinical settings and that survival is observed in a proportion of patients, though the wide outcome ranges preclude precise prognostication for individual patients. Clinicians must also weigh the ethical dimension of this decision: proceeding with PCI allocates substantial resources at a time when the patient’s neurological prognosis remains fundamentally uncertain, and the possibility of survival with severe neurological disability cannot be excluded.

The selection characteristics described in this review may help inform, but should not rigidly define, patient selection criteria. The predominance of younger patients, shockable rhythms, and moderate low-flow times in the included studies likely reflects a combination of clinical judgement and institutional protocols that have evolved through experience.

#### 4.3.2. Standardizing Reporting for Future Research

Perhaps the most actionable implication of this review is the need for standardized documentation of ROSC status at key clinical timepoints in future ECPR studies. The observation that fewer than half of included studies explicitly documented ROSC status at PCI initiation represents a substantial reporting gap. We propose the following minimum reporting elements for future ECPR-PCI studies: (1) whether sustained ROSC was achieved prior to catheterization laboratory arrival, (2) the hemodynamic support status (including anticoagulation regimen) at the time of coronary intervention, (3) the temporal relationship between ECMO initiation, catheterization laboratory arrival, and PCI, and (4) the vascular access strategy for coronary catheterization relative to ECMO cannulation sites.

### 4.4. Strengths and Limitations

#### 4.4.1. Strengths

This review has several methodological strengths. First, it is the first systematic effort to characterize the specific phenotype of PCI without sustained ROSC under ECPR, addressing a clinically relevant gap. Second, the three-axis framework (Selection, Feasibility, and Outcomes) provides an organized structure for synthesizing heterogeneous data. Third, the explicit phenotype classification system with transparent evidence documentation allows readers to assess the basis for each study’s inclusion. Fourth, the study design—descriptive synthesis without meta-analysis—is appropriate for the current evidence landscape, avoiding the false precision of pooled estimates from heterogeneous populations. Fifth, protocol registration in PROSPERO and adherence to PRISMA 2020 guidelines enhance transparency and reproducibility.

#### 4.4.2. Limitations

Several limitations warrant consideration, organized here along the major bias domains of ROBINS-I to make the methodological constraints explicit rather than summarily acknowledged.

**Confounding by indication (ROBINS-I D1) and selection bias (D2) are the dominant methodological constraints.** All included studies were observational, predominantly retrospective, and subject to confounding by indication for PCI (no study randomly assigned PCI while on ECMO without sustained ROSC). Selection into ECPR itself is a substantial filter—restricted to patients judged to have a potentially reversible cause, favourable baseline status, and acceptable low-flow time—which produces systematic differences between the studied population and the broader refractory cardiac arrest population. **Immortal time bias** is a particular concern: patients who die before reaching the catheterization laboratory cannot by definition receive PCI, so cohorts defined by “patients who underwent PCI during ECPR” over-represent early survivors. **Survivorship bias** further affects outcomes measured after ECMO cannulation, since patients who die during cannulation are excluded from several cohorts. **Centre effects** are evident in the wide range of PCI rates (24–100%) and regional variation, implying that outcome differences between cohorts are at least partially driven by institutional protocol and expertise rather than patient-level characteristics. Our ROBINS-I assessment rated 9 of 13 DEFINITE studies at serious overall risk of bias driven predominantly by D1 and D2, confirming that these constraints are structural to the current evidence base.

**Phenotype-classification uncertainty (D3).** Classification required inference from clinical workflows in 14 of 27 studies (PROBABLE category), introducing potential misclassification. The DEFINITE-primary/PROBABLE-supportive structure, combined with the DEFINITE-only sensitivity analysis, quantifies this uncertainty but does not eliminate it.

**Missing data (D5) and outcome measurement heterogeneity (D6).** Outcome definitions varied across studies as follows: survival endpoints included hospital discharge, ICU discharge, and 30-day survival, while favourable neurological outcome was assessed at hospital discharge, 30 days, ICU discharge, or other timepoints. This heterogeneity limits direct comparability. Two clinically important variables—anticoagulation strategy during ECMO-supported PCI and vascular access site selection—were not systematically reported in any included study.

**Selection of reported results (D7) and publication bias.** Publication bias may favour studies with favourable outcomes, potentially skewing the observed envelope; this risk is inherent to descriptive reviews and cannot be assessed with standard funnel-plot methods in the absence of pooled estimates.

**Overlap between registries.** SAVE-J II and ELSO-linked studies may contain up to ~8.5% patient-level overlap. The pre-specified exclude-overlap sensitivity analysis (Section 3.5) produced concordant findings, but residual overlap cannot be fully excluded.

**Review-team and AI-assistance considerations.** This review was conducted by a small team that used AI-assisted drafting for title/abstract screening recommendations and for phenotype-classification drafts; all decisions were confirmed by human reviewers, and the ROBINS-I assessment was performed by a single reviewer (Y.H.C.). This workflow differs from traditional dual-independent screening and dual-independent risk-of-bias assessment; we did not compute κ because the verification process was not fully independent, and we adopted the DEFINITE-primary framework and sensitivity analyses in lieu of formal inter-rater reliability.

**Generalisability.** The predominantly single-centre nature of many included studies and geographic concentration in Japan and the United States, together with English-language full-text restriction, may limit generalisability to other healthcare systems.

### 4.5. Future Directions

Several directions for future research emerge from this review. First, prospective registries with standardized phenotype documentation—explicitly reporting ROSC status at the time of coronary intervention—would enable more precise characterization of this population. Second, propensity-matched or target-trial-emulation analyses could provide additional observational evidence on outcomes between patients who undergo PCI without sustained ROSC and those in whom PCI is deferred; such analyses would describe observed outcome differences but would remain subject to unmeasured confounding and therefore should not be interpreted as estimates of treatment effectiveness. Third, studies examining the hemodynamic adequacy of ECMO-supported perfusion during PCI, including coronary flow dynamics and end-organ perfusion markers, could inform procedural optimization. Fourth, international collaborative efforts to harmonize ECPR protocols and outcome reporting—building on existing frameworks such as the Utstein guidelines [8] and subsequent ECPR-specific reporting recommendations—would facilitate meaningful cross-study comparisons.

### 4.6. Conclusions

This descriptive systematic review characterized the phenotype of PCI performed during ECPR without sustained ROSC by pre-classifying the literature into a DEFINITE primary cohort (13 studies, *N* = 3320) and a PROBABLE supportive cohort (14 studies, *N* = 9562), with exclude-overlap and DEFINITE-only sensitivity analyses supporting the robustness of the descriptive envelope. PCI is **technically feasible** under ECMO support, with post-procedural TIMI 3 flow reported in 62.4–84.0% of reporting studies—approaching, though falling below, the rates observed in conventional primary PCI. In the DEFINITE cohort, unweighted median survival to discharge was 30.3% (IQR 26.5–40.8) and favourable neurological outcome 33.5% (IQR 16.8–45.8); ranges were wide (survival 21.0–69.0%; CPC 1–2 10.4–92.0%) and were driven by substantial heterogeneity in patient selection, institutional protocols, and endpoint definitions. ROBINS-I rated 9 of 13 DEFINITE studies at serious overall risk of bias, predominantly from confounding by indication and selection, reinforcing that these findings describe an observed clinical practice rather than quantifying a treatment effect. **No inference regarding comparative effectiveness of PCI under these conditions is possible from the available observational evidence.** The practice is widespread but insufficiently standardized in ROSC reporting; the most immediate priority for future ECPR research is the adoption of standardized documentation of ROSC status at the time of coronary intervention.

## Figures and Tables

**Table 1:** Selection profile—who gets selected? Axis 1: Selection characteristics of patients who underwent PCI without sustained ROSC under ECPR. Data presented as reported by individual studies. Low-flow definition markers: a = collapse-to-ECMO; b = call-to-ECMO; c = CPR duration; d = total professional CPR; e = other/unspecified. Dagger (†) denotes PROBABLE classification.

Study	Country	Design	Period	N	Age	Male %	OHCA %	Shockable %	Low-Flow (min)	STEMI %
Nakashima (2025) [12]	Japan	Reg	January 2013 to December 2018	251	61 (51, 67)	89.2	100.0	76.5	56 (47, 68) b	NR
Crespo-Diaz (2024) [13]	USA	RC	December 2015 to June 2022	289	59 ± 16	80.0	100.0	100.0	56 ± 19 e	NR
Nishimura (2024) [14]	Japan	Reg	January 2013 to December 2018	877	62 (52, 69)	89.5	100.0	73.4	54 (45, 65) a	NR
Nakashima (2023) [15]	Japan	Reg	January 2013 to December 2018	624	NR	NR	100.0	74.5	56 (46, 69) b	NR
Kawakami (2022) [16]	Japan	Reg	June 2014 to December 2017	695	59 (47, 68)	84.2	100.0	72.4	56 (47, 68) b	NR
Hryniewicz (2021) [17]	USA	RC	January 2012 to December 2017	26	59 ± 11	65.0	27.0	65.0	51 (22, 70)	NR
Bartos (2020) [18]	USA	PC	December 2019 to April 2020	45	57 ± 14	79.3	100.0	100.0	52.2 ± 17.0 d	NR
Boudoulas (2020) [19]	USA	RC	August 2017 to June 2019	16	56.4 ± 14.1	93.8	100.0	100.0	44.2 ± 9.2	NR
Duerschmied (2020) [20]	Germany	Reg	May 2011 to May 2019	252	59 ± 14.3	73.8	44.4	47.6	52 (10–150) a	NR
Cesana (2018) [21]	Italy	RC	January 2011 to October 2015	63	59 ± 10	87.0	73.0	65.0	56 ± 24 c	68.0
Dennis (2017) [22]	Australia	RC	2009 to 2016	37	54 (47, 58)	73.0	32.0	51.0	45 (30, 70) a	NR
Kuroki (2017) [23]	Japan	RC	January 2005 to June 2016	119	63.2 ± 11.8	91.0	31.0	33.0	34.2 ± 16.1 a	61.0
Stub (2015) [24]	Australia	PC	32-month period	26	52 (38, 60)	77.0	42.3	73.0	56 (40, 85) a	34.6
Alhuneafat (2025) † [25]	International	Reg	January 2020 to December 2022	576	58.6 (49.3–64.5)	83.3	100.0	75.4	56 (50.0–70.0) c	34.1
Righetti (2025) † [26]	Italy	RC	January 2011 to December 2022	129	56 (48–62)	86.8	100.0	72.9	67 (58.5–75.0) e	NR
Fu (2024) † [27]	Taiwan	RC	2010 to 2022	215	60.1 (51.3–67.9)	89.7	49.4	68.9	NR	NR
Takeuchi (2024) † [28]	Japan	RC	January 2009 to December 2020	50	57.6	90.0	100.0	86.0	NR	92.0
Kim (2023) † [29]	South Korea	Reg	January 2013 to December 2020	564	56.4 (46–64.5)	82.1	100.0	58.5	46 (35–63) b	NR
Tonna (2022) † [30]	International	Reg	2008 to 2019	7488	55 (44–64)	68.0	NR	NR	NR	NR
Chico-Carballas (2021) † [31]	Spain	CS	November 2017 to November 2018	7	62 (40–68)	57.1	0.0	71.4	55 (36–63) a	57.1
Porto (2021) † [32]	Italy	RC	January 2010 to December 2015	21	NR	NR	NR	NR	NR	71.0
Radsel (2021) † [33]	Slovenia	RC	June 2010 to February 2020	52	52 ± 12	82.7	42.3	NR	NR	NR
Lamhaut (2018) † [34]	France	PC	October 2014 to December 2016	74	54 ± 12.2	81.0	100.0	66.0	78 ± 26 c	NR
Haas (2017) † [35]	International	Reg	2010 to 2016	217	52 (45–62)	73.0	100.0	NR	NR	NR
Sakai (2014) † [36]	Japan	Reg	March 2008 to December 2008	35	60.2 ± 13.4	85.7	100.0	100.0	31.2 ± 10.5 a	NR
Sakamoto (2012) † [37]	Japan	RC	January 2000 to December 2010	98	72 ± 12	66.3	NR	23.5	25.5 ± 22.4 a	NR
Chen (2006) † [38]	Taiwan	RC	1994 to 2003	36	57 ± 10	91.7	NR	NR	NR	NR

**Abbreviations:** RC, retrospective cohort; PC, prospective cohort; Reg, registry; CS, case series; NR, not reported. Data presented as median [IQR] or mean ± SD as reported in original studies.

**Table 2:** Procedural feasibility—can it actually be done? Axis 2: Procedural feasibility of PCI under ECMO support without sustained ROSC. Dagger (†) denotes PROBABLE classification. NR = not reported.

Study	*N*	*N* (PCI)	Angio %	PCI %	TIMI 3 %	IABP %	Bleeding *n*	Limb Isch *n*
Nakashima (2025) [12]	251	251	100.0	100.0	69.7	89.6	NR	NR
Crespo-Diaz (2024) [13]	289	165	100.0	57.1	NR	NR	NR	NR
Nishimura (2024) [14]	877	687	NR	78.3	NR	80.0	NR	NR
Nakashima (2023) [15]	624	624	NR	NR	80.0	NR	NR	NR
Kawakami (2022) [16]	695	285	71.9	41.0	NR	59.4	NR	NR
Hryniewicz (2021) [17]	26	—	NR	NR	NR	46.0	18	NR
Bartos (2020) [18]	45	22	100.0	48.9	NR	NR	4	0
Boudoulas (2020) [19]	16	6	93.8	37.5	NR	NR	NR	NR
Duerschmied (2020) [20]	252	159	74.6	63.1	NR	NR	NR	NR
Cesana (2018) [21]	63	—	100.0	NR	NR	38.0	NR	NR
Dennis (2017) [22]	37	9	54.0	24.0	NR	5.0	14	7
Kuroki (2017) [23]	119	119	100.0	100.0	84.0	91.0	NR	NR
Stub (2015) [24]	26	11	80.8	42.3	NR	NR	18	1
Alhuneafat (2025) † [25]	576	138	41.0	24.0	NR	NR	NR	NR
Righetti (2025) † [26]	129	94	94.6	72.9	62.4	NR	NR	NR
Fu (2024) † [27]	215	175	100.0	81.4	NR	75.0	NR	NR
Takeuchi (2024) † [28]	50	50	100.0	100.0	NR	NR	NR	7
Kim (2023) † [29]	564	211	56.6	37.4	NR	NR	NR	NR
Tonna (2022) † [30]	7488	—	6.9	NR	NR	NR	NR	NR
Chico-Carballas (2021) † [31]	7	3	71.4	42.9	NR	NR	1	0
Porto (2021) † [32]	21	21	100.0	100.0	NR	86.0	NR	4
Radsel (2021) † [33]	52	24	73.1	46.2	NR	42.3	7	16
Lamhaut (2018) † [34]	74	49	100.0	66.0	NR	NR	NR	NR
Haas (2017) † [35]	217	57	NR	26.3	NR	7.4	68	24
Sakai (2014) † [36]	35	14	60.0	40.0	NR	NR	NR	NR
Sakamoto (2012) † [37]	98	92	99.0	93.9	70.7	95.9	23	7
Chen (2006) † [38]	36	7	NR	30.6	NR	100.0	NR	NR

**Abbreviations**: Angio, coronary angiography; PCI, percutaneous coronary intervention; TIMI, thrombolysis in myocardial infarction; IABP, intra-aortic balloon pump; NR, not reported; —, not specified separately.

**Table 3:** Outcome envelope—what can be expected? (exploratory) Axis 3: Outcome envelope observed in patients who underwent PCI without sustained ROSC under ECPR. This table provides a range of observed outcomes, not predicted values. Dagger (†) denotes PROBABLE classification. Asterisk (*) denotes ICU discharge endpoint. NR = not reported.

Study	*N*	Surv %	Endpoint	Neuro Good %	Neuro Timing	Weaning %	ECMO Duration	Post-PCI ROSC %
Nakashima (2025) [12]	251	21.1	Hosp	10.4	Discharge	27.1	NR	64.9
Crespo-Diaz (2024) [13]	289	NR	—	40.5	Discharge	NR	NR	NR
Nishimura (2024) [14]	877	30.3	Hosp	15.1	30 d/discharge	NR	4.0 d [3, 5]	NR
Nakashima (2023) [15]	624	33.3	Hosp	17.3	Discharge	NR	NR	NR
Kawakami (2022) [16]	695	NR	—	11.1	30 d	NR	NR	NR
Hryniewicz (2021) [17]	26	69.0	Hosp	65.0	Discharge	NR	4.5 d [2.9, 6.1]	NR
Bartos (2020) [18]	45	46.6	Hosp	43.1	Discharge	NR	4.2 d ± 1.5	NR
Boudoulas (2020) [19]	16	25.0	Hosp	25.0	Discharge	NR	3.8 d ± 2.2	NR
Duerschmied (2020) [20]	252	29.4	Hosp	NR	—	NR	NR	NR
Cesana (2018) [21]	63	21.0	Hosp	92.0	Discharge	33.0	6 d ± 4	NR
Dennis (2017) [22]	37	35.0	Hosp	35.0	Discharge	41.0	3 d [1, 6]	NR
Kuroki (2017) [23]	119	28.0	Hosp	32.0	30 d	43.0	3.0 d ± 2.1	NR
Stub (2015) [24]	26	53.8	Hosp	53.8	Discharge	54.2	2 d [1, 5]	NR
Alhuneafat (2025) † [25]	576	18.1	Hosp	NR	—	NR	NR	NR
Righetti (2025) † [26]	129	17.1 *	ICU	13.2	ICU discharge	NR	NR	NR
Fu (2024) † [27]	215	NR	—	NR	—	NR	NR	NR
Takeuchi (2024) † [28]	50	NR	—	30.0	30 d	NR	NR	NR
Kim (2023) † [29]	564	13.8	Hosp	9.8	Discharge	NR	1.0 d [0.2–3.2]	NR
Tonna (2022) † [30]	7488	29.0	Hosp	NR	—	NR	NR	NR
Chico-Carballas (2021) † [31]	7	42.9	Hosp	42.9	Discharge	NR	5 d [1–7,9]	NR
Porto (2021) † [32]	21	19.0	Hosp	NR	—	38.0	NR	NR
Radsel (2021) † [33]	52	28.8	Hosp	28.8	Discharge	42.3	3.4 d ± 5.7	NR
Lamhaut (2018) † [34]	74	NR	—	31.0	ICU discharge	NR	NR	NR
Haas (2017) † [35]	217	27.6	Hosp	NR	—	NR	2.0 d [0.7–3.9]	NR
Sakai (2014) † [36]	35	NR	—	28.6	1 month	NR	NR	NR
Sakamoto (2012) † [37]	98	32.7	Hosp	NR	—	55.1	2.9 d ± 2.6	NR
Chen (2006) † [38]	36	33.3	Hosp	30.6	Follow-up	69.4	4.5 d ± 3.2	NR
**Observed Range**		**13.8–69.0**		**9.8–92.0**		**27.1–69.4**	**1.0–6.0 d**	**64.9**

**Critical Interpretation Note:** > these ranges represent **what has been observed** in heterogeneous real-world cohorts, not **what should be expected** for individual patients. Actual outcomes are highly dependent on selection criteria (Axis 1) and procedural success (Axis 2). **Note:** Survival endpoint varies across studies (hospital discharge, ICU discharge, 30-day); the observed range row reflects the full spectrum across all endpoint definitions. Direct comparison of individual study rates should account for endpoint heterogeneity. **Abbreviations:** Neuro, neurological outcome (CPC 1–2); CPC, cerebral performance category; ECMO, extracorporeal membrane oxygenation; ROSC, return of spontaneous circulation; d, days; Hosp, hospital discharge; NR, not reported. **Label: exploratory analysis.**

## Data Availability

The data supporting the findings of this systematic review are available in the article and its Supplementary Materials. The complete extraction dataset (27 studies × 93 variables) is provided as Table S8 (a separate Microsoft Excel file accompanying the Supplementary Materials). Search strategies, JBI and ROBINS-I risk-of-bias appraisals, excluded study lists, phenotype classification evidence, the PRISMA 2020 checklist, and sensitivity analyses are provided in Tables S1–S7. The study protocol is registered in PROSPERO (CRD420251252255).

## Supplementary Materials

The following supporting information can be downloaded at: https://www.mdpi.com/article/10.3390/jcm15124422/s1, Table S1: Search Strategies. Table S2: JBI Critical Appraisal (All 27 Studies). Table S3: Excluded Studies with Reasons. Table S4: Phenotype Classification Evidence. Table S5: PRISMA 2020 Checklist. Table S6: Sensitivity Analyses. Table S7: ROBINS-I Risk-of-Bias Assessment (13 DEFINITE Studies). Table S8: Complete Extraction Dataset (27 Studies × 93 Variables; provided as a separate Microsoft Excel file). Figure S1: JBI Critical Appraisal Summary Bar Chart (All 27 Studies). Figure S2: Shockable Rhythm vs. Survival (Exploratory). Figure S3: Combined Outcome Panel.
