# Supplementary material for: Percutaneous Coronary Intervention Without Sustained Return of Spontaneous Circulation Under Extracorporeal Cardiopulmonary Resuscitation: A Phenotype-Oriented Descriptive Systematic Review
Source: J Clin Med. 2026 Jun 7;15(12):4422. doi: 10.3390/jcm15124422 (PMC13302444; doi:10.3390/jcm15124422)
Supplement: Supplementary file 1 [file jcm-15-04422-s001.zip › jcm-4234957-supplementary TabS1-7,FigS1-3.pdf]

Table S1. Full Electronic Search Strategies

Systematic Review: PCI Without Sustained ROSC Under ECPR — A Phenotype-Oriented Descriptive Systematic Review

All databases were searched from January 1, 2000 to present. No language restrictions were applied at the search stage. The search strategy was designed to prioritize sensitivity, as the phenotype of interest (PCI without sustained ROSC) is not directly searchable via controlled vocabulary or keywords.

---

1. MEDLINE via PubMed

Date Range: 2000-01-01 to present **Restrictions:** Humans

Search String

```
(
  ("Extracorporeal Membrane Oxygenation"[Mesh] OR "Cardiopulmonary Resu
scitation"[Mesh]
   OR ECPR[tiab] OR "extracorporeal cardiopulmonary resuscitation"[tiab]
   OR "extracorporeal CPR"[tiab] OR E-CPR[tiab] OR "VA-ECMO"[tiab]
   OR "VA ECMO"[tiab] OR "venoarterial ECMO"[tiab]
   OR "venoarterial extracorporeal membrane oxygenation"[tiab]
   OR "ECMO resuscitation"[tiab] OR "mechanical circulatory support"[ti
ab]
   OR "extracorporeal life support"[tiab] OR ECLS[tiab])
  AND
  ("Percutaneous Coronary Intervention"[Mesh] OR "Coronary Angiography"
[Mesh]
   OR "Cardiac Catheterization"[Mesh] OR PCI[tiab]
   OR "percutaneous coronary intervention"[tiab] OR "coronary intervent
ion"[tiab]
   OR "coronary angiography"[tiab] OR "coronary angiogram"[tiab]
   OR "cardiac catheterization"[tiab] OR "cardiac catheterisation"[tiab]
   OR "coronary revascularization"[tiab] OR "coronary revascularisation
"[tiab]
   OR angioplasty[tiab] OR "coronary stent"[tiab] OR "coronary stenting
"[tiab]
   OR "invasive coronary"[tiab])
  AND
  ("Heart Arrest"[Mesh] OR "Out-of-Hospital Cardiac Arrest"[Mesh]
   OR "cardiac arrest"[tiab] OR "heart arrest"[tiab]
   OR "cardiopulmonary arrest"[tiab] OR "circulatory arrest"[tiab]
   OR OHCA[tiab] OR "out-of-hospital cardiac arrest"[tiab]
   OR "out of hospital cardiac arrest"[tiab] OR IHCA[tiab]
   OR "in-hospital cardiac arrest"[tiab] OR "in hospital cardiac arrest
```

```
"[tiab]
   OR "sudden cardiac arrest"[tiab] OR "refractory cardiac arrest"[tiab]
   OR "refractory arrest"[tiab] OR "refractory VF"[tiab]
   OR "refractory VT"[tiab] OR "refractory ventricular fibrillation"[ti
ab]
   OR "refractory ventricular tachycardia"[tiab] OR "resuscitation"[tia
b]
   OR CPR[tiab] OR ROSC[tiab] OR "return of spontaneous circulation"[ti
ab])
)
AND
("2000/01/01"[Date - Publication] : "3000"[Date - Publication])
AND
(Humans[Mesh])
```

Pilot Search Yield: ~586 citations

---

2. Embase via Ovid

Date Range: 2000-01-01 to present **Restrictions:** Humans, Adults (18+)

Search String

```
1. exp extracorporeal membrane oxygenation/ or exp heart resuscitation/
2. (ECPR or "extracorporeal cardiopulmonary resuscitation"
   or "extracorporeal CPR" or E-CPR or "VA-ECMO" or "VA ECMO"
   or "venoarterial ECMO"
   or "venoarterial extracorporeal membrane oxygenation"
   or "ECMO resuscitation" or "mechanical circulatory support"
   or "extracorporeal life support" or ECLS).ti,ab,kw.
3. 1 or 2
4. exp percutaneous coronary intervention/ or exp coronary angiography/
   or exp heart catheterization/
5. (PCI or "percutaneous coronary intervention" or "coronary interventi
on"
   or "coronary angiography" or "coronary angiogram"
   or "cardiac catheterization" or "cardiac catheterisation"
   or "coronary revascularization" or "coronary revascularisation"
   or angioplasty or "coronary stent" or "coronary stenting"
   or "invasive coronary").ti,ab,kw.
6. 4 or 5
7. exp heart arrest/ or exp out of hospital cardiac arrest/
8. ("cardiac arrest" or "heart arrest" or "cardiopulmonary arrest"
   or "circulatory arrest" or OHCA or "out-of-hospital cardiac arrest"
   or "out of hospital cardiac arrest" or IHCA
   or "in-hospital cardiac arrest" or "in hospital cardiac arrest"
   or "sudden cardiac arrest" or "refractory cardiac arrest"
   or "refractory arrest" or "refractory VF" or "refractory VT"
   or "refractory ventricular fibrillation"
```

```
   or "refractory ventricular tachycardia" or resuscitation
   or CPR or ROSC or "return of spontaneous circulation").ti,ab,kw.
9. 7 or 8
10. 3 and 6 and 9
11. limit 10 to (human and yr="2000-Current")
12. limit 11 to (adult <18 to 64 years> or aged <65+ years>)
```

---

3. Cochrane Library (CENTRAL)

Date Range: January 2000 to January 2026 (search date) **Language:** All languages

Search String

```
#1  MeSH descriptor: [Extracorporeal Membrane Oxygenation] explode all
trees
#2  MeSH descriptor: [Cardiopulmonary Resuscitation] explode all trees
#3  (ECPR OR "extracorporeal cardiopulmonary resuscitation"
     OR "extracorporeal CPR" OR "E-CPR" OR "VA-ECMO" OR "VA ECMO"
     OR "venoarterial ECMO"
     OR "venoarterial extracorporeal membrane oxygenation"
     OR "ECMO resuscitation" OR "mechanical circulatory support"
     OR "extracorporeal life support" OR ECLS):ti,ab,kw
#4  #1 OR #2 OR #3
#5  MeSH descriptor: [Percutaneous Coronary Intervention] explode all t
rees
#6  MeSH descriptor: [Coronary Angiography] explode all trees
#7  MeSH descriptor: [Cardiac Catheterization] explode all trees
#8  (PCI OR "percutaneous coronary intervention" OR "coronary intervent
ion"
     OR "coronary angiography" OR "coronary angiogram"
     OR "cardiac catheterization" OR "cardiac catheterisation"
     OR "coronary revascularization" OR "coronary revascularisation"
     OR angioplasty OR "coronary stent" OR "coronary stenting"
     OR "invasive coronary"):ti,ab,kw
#9  #5 OR #6 OR #7 OR #8
#10 MeSH descriptor: [Heart Arrest] explode all trees
#11 MeSH descriptor: [Out-of-Hospital Cardiac Arrest] explode all trees
#12 ("cardiac arrest" OR "heart arrest" OR "cardiopulmonary arrest"
     OR "circulatory arrest" OR OHCA OR "out-of-hospital cardiac arrest
"
     OR "out of hospital cardiac arrest" OR IHCA
     OR "in-hospital cardiac arrest" OR "in hospital cardiac arrest"
     OR "sudden cardiac arrest" OR "refractory cardiac arrest"
     OR "refractory arrest" OR "refractory VF" OR "refractory VT"
     OR "refractory ventricular fibrillation"
     OR "refractory ventricular tachycardia" OR resuscitation
     OR CPR OR ROSC OR "return of spontaneous circulation"):ti,ab,kw
#13 #10 OR #11 OR #12
```

```
#14 #4 AND #9 AND #13
#15 #14 with Cochrane Library publication date Between Jan 2000 and Jan
 2026
```

Search Concept Structure

All three database searches used the same three-concept Boolean AND structure:

Concept	Description	Controlled Vocabulary	Free-Text Terms
**1. ECPR / VA-ECMO**	Extracorporeal cardiopulmonary resuscitation	MeSH: Extracorporeal Membrane Oxygenation; Cardiopulmonary Resuscitation. Emtree: extracorporeal membrane oxygenation; heart resuscitation	ECPR, extracorporeal CPR, E-CPR, VA-ECMO, venoarterial ECMO, ECMO resuscitation, mechanical circulatory support, extracorporeal life support, ECLS
**2. PCI / Coronary Intervention**	Percutaneous coronary intervention and coronary angiography	MeSH: Percutaneous Coronary Intervention; Coronary Angiography; Cardiac Catheterization. Emtree: percutaneous coronary intervention; coronary angiography; heart catheterization	PCI, coronary intervention, coronary angiography, coronary angiogram, cardiac catheterization/catheterisation, coronary revascularization/revascularisation, angioplasty, coronary stent/stenting, invasive coronary
**3. Cardiac Arrest**	Cardiac arrest including refractory arrest	MeSH: Heart Arrest; Out-of-Hospital Cardiac Arrest. Emtree: heart arrest; out of	cardiac arrest, heart arrest, cardiopulmonary arrest, circulatory arrest, OHCA,

Concept	Description	Controlled Vocabulary	Free-Text Terms
		hospital cardiac arrest	IHCA, sudden/refractory cardiac arrest, refractory VF/VT, resuscitation, CPR, ROSC

Deduplication Strategy

Records were exported from each database and deduplicated in the following priority order: 1. PubMed (primary; PMID used as unique identifier) 2. Embase (secondary; DOI cross-reference) 3. Cochrane CENTRAL (tertiary)

Deduplication was performed using reference management software with DOI and title matching.

Table S2. JBI Critical Appraisal — All 27 Included Studies

Reporting-quality appraisal using the JBI Critical Appraisal Checklists (case-series tool for single-arm descriptive studies; cohort tool [2024 revision] for studies with comparison groups). Overall concern categorised as Low (≥75% of applicable items scored "Yes"), Moderate (50–74%), or High (<50%). This appraisal characterises *reporting quality* and is provided as a supportive reference; the primary risk-of-bias assessment is the ROBINS-I appraisal of the 13 DEFINITE studies (Table S7; main-text Figure 6).

Study	Classification	JBI Tool	Overall Concern
Nakashima (2025)	DEFINITE	Cohort (2024)	Low concern
Crespo-Diaz (2024)	DEFINITE	Cohort (2024)	Low concern
Nishimura (2024)	DEFINITE	Cohort (2024)	Low concern
Nakashima (2023)	DEFINITE	Cohort (2024)	Low concern
Kawakami (2022)	DEFINITE	Cohort (2024)	Low concern
Hryniewicz (2021)	DEFINITE	Case series	Low concern
Bartos (2020)	DEFINITE	Case series	Low concern
Boudoulas (2020)	DEFINITE	Case series	Low concern

Study	Classification	JBI Tool	Overall Concern
Duerschmied (2020)	DEFINITE	Case series	Low concern
Cesana (2018)	DEFINITE	Cohort (2024)	Moderate concern
Dennis (2017)	DEFINITE	Case series	Low concern
Kuroki (2017)	DEFINITE	Case series (2024)	Low concern
Stub (2015)	DEFINITE	Case series	Low concern
Alhuneafat (2025)	PROBABLE	Cohort (2024)	Low concern
Righetti (2025)	PROBABLE	Case series	Low concern
Fu (2024)	PROBABLE	Cohort (2024)	Low concern
Takeuchi (2024)	PROBABLE	Cohort (2024)	Moderate concern
Kim (2023)	PROBABLE	Cohort (2024)	Low concern
Tonna (2022)	PROBABLE	Cohort (2024)	Low concern
Chico-Carballas (2021)	PROBABLE	Case series	Low concern
Porto (2021)	PROBABLE	Case series	Low concern
Radsel (2021)	PROBABLE	Case series	Low concern
Lamhaut (2018)	PROBABLE	Case series	Low concern
Haas (2017)	PROBABLE	Case series	Moderate concern
Sakai (2014)	PROBABLE	Case series	Low concern
Sakamoto (2012)	PROBABLE	Case series	Low concern
Chen (2006)	PROBABLE	Case series	Low concern

Distribution: 24 studies (88.9%) Low concern, 3 (11.1%) Moderate concern, 0 High concern. A summary bar chart is shown in Figure S1.

Table S3. Studies Excluded at Full-Text Screening With Reasons

Systematic Review: PCI Without Sustained ROSC Under ECPR — A Phenotype-Oriented Descriptive Systematic Review

Studies were excluded after full-text review based on the following criteria: case report/series with <5 patients, not original research (review/editorial/letter/guideline), not ECPR population, no PCI/coronary angiography data, conference abstract only, non-English without accessible full text, duplicate, pediatric population, animal study, PDF unavailable, or inability to classify ROSC-at-PCI phenotype.

RecordID	First Author	Year	Exclusion Reason
24	Nishimura T	2025	No PCI/angiography data reported; focused on public assistance influence on outcomes (SAVE-J II secondary analysis)
43	Gelmann D	2025	Simulation study; not original clinical research with real patients
71	Kalra R	2025	No PCI/coronary angiography data; hemodynamic/physiological study focused on LV energetics during ECPR
89	Mazzeffi M	2024	No PCI/coronary angiography data; ELSO registry analysis focused on cannulation location and IHCA
118	Soriano M	2024	Case report (N=1); Kounis syndrome with refractory cardiac arrest
144	Sun P	2023	Systematic review/meta-analysis; not original research
145	Voß F	2024	No granular PCI/coronary angiography data; cardiac arrest center accreditation study

RecordID	First Author	Year	Exclusion Reason
164	Puolakka T	2023	PDF unavailable (BMJ paywall); no PMC/free access
165	Gama M	2023	Case report (N=1); arrhythmogenic LV cardiomyopathy
192	Tong H	2023	No PCI-specific data; study focused on early CT scan after ECPR; PCI used only as matching covariate
236	Hong S	2022	No PCI/coronary angiography data; focused on optimal PaCO2/PaO2 after ECPR
272	Catalano MA	2022	Cannot determine ROSC status at time of PCI from ICD codes (NIS administrative database); insufficient granularity for phenotype classification
281	Nair SG	2022	Case report (N=1); single OHCA patient with LAD occlusion
319	Watanabe T	2020	Conference abstract only (1-page ESC Congress abstract); insufficient data for phenotype classification

RecordID	First Author	Year	Exclusion Reason
326	Hifumi T	2021	Institutional-level survey (36 centers); no individual patient outcome data
337	George N	2022	Case report (N=1); not original research with >=5 patients
361	Ohlemacher F	2020	Non-English article (German language)
386	Meyer AJ	2020	Case report (N=1); fulminant myocarditis
392	Fernando SM	2020	Educational/review article; not original research
405	Kapur NK	2021	Case report (N=1); post-cardiac surgery arrest, not ECPR context
440	Bartos JA	2020	Duplicate of RecordID 343 (same PDF, identical MD5 hash)
445	Sammut MA	2020	Case report (N=1); post-aortic valve replacement cardiac arrest
456	Sugiyama K	2019	Pediatric case report (13-year-old); age <18 and N=1
524	Bougle A	2019	Not ECPR phenotype; post-cardiac arrest shock (patients had ROSC first, then developed

RecordID	First Author	Year	Exclusion Reason
			shock); ECLS initiated median 9h after cardiac arrest
654	Bhandary SP	2017	Review/narrative article; not original research
667	Hutin A	2016	Animal study (porcine model, N=14 swine); not human clinical research
680	Han KS	2017	Duplicate PDF (same content as RecordID 654); review article
695	Huang L	2016	Non-English article (Chinese); correct PDF not available
742	Song KH	2015	Pediatric case report; excluded by age criteria
751	Dreher M	2016	Non-English article (German); correct PDF not available
762	Ozyilmaz I	2015	Pediatric case report; hasPCI=false
765	Lazzeri C	2016	Review/editorial article; not original research
825	Rassaf T	2014	Case report (N=1); perfect phenotype match but excluded per protocol (<5 patients)
856	Shin TG	2013	Cannot isolate PCI subgroup outcomes; cannot determine ROSC

RecordID	First Author	Year	Exclusion Reason
			status at PCI initiation (E-CPR vs C-CPR comparison)
896	Chung JW	2012	Case report (N=2); <5 patients
897	Goswami S	2013	Case report (N=2); focused on brain death determination during ECPR
922	Wu MY	2012	Mixed cardiogenic shock and cardiac arrest population; cannot isolate >=5 ECPR patients who received PCI
974	Puehler T	2010	Case report (N=1); letter describing single patient with LM occlusion during PCI
1052	Shin JS	2007	Systematic review/literature review; not original research
1123	Kim D	2025	Letter/correspondence; not original research
1155	Wengenmayer T	2025	No PCI/coronary angiography data; VA-ECMO registry focused on ECMO outcomes
1158	Abi Abdallah G	2025	Case report (N=1)
1173	Hu C	2025	Case report (N=1); single patient with AMI cardiac arrest treated with thrombolysis +

RecordID	First Author	Year	Exclusion Reason
			ECPR
1198	Smalcova J	2025	No PCI/coronary angiography data; focused on organ donation outcomes after ECPR
1228	Nakamura K	2025	Case report (N=1); Impella complication report
1261	Inoue F	2025	No PCI data; focused on non-cardiac etiologies (intracranial hemorrhage and acute aortic syndromes)
1327	Mangner N	2024	Conference abstract on Impella in cardiogenic shock; not ECPR for cardiac arrest
1378	Leung KHB	2024	Geospatial modelling study; no clinical patient data or PCI outcomes
1404	Gregers E	2024	No coronary angiography or PCI data; functional outcome study (return to work); cannot classify ROSC-at-PCI phenotype
1420	Gregers E	2024	No coronary angiography or PCI data; HRQoL/cognition

RecordID	First Author	Year	Exclusion Reason
			outcome study; cannot classify ROSC-at-PCI phenotype
1494	Morrow DA	2023	Editorial/perspective; not original research
1551	Watanabe M	2023	Conference abstract collection (multiple unrelated abstracts); not a single original research article
1634	Liu Y	2022	Letter/commentary; no granular PCI data
1640	Tschope C	2022	Not ECPR for cardiac arrest; focused on Impella/biopsy in acute heart failure
1678	Radsel P	2022	Not ECPR population; AMI-cardiogenic shock patients; VA-ECMO mentioned only as covariate
1725	Pineton De Chambrun M	2022	Editorial/commentary; not original research
1744	Mendes JJ	2022	Case report (N=1)
1750	Scherer C	2022	Cardiogenic shock focus; not cardiac arrest
1769	Williams C	2022	Case report (N=1); DRESS syndrome fulminant myocarditis
1788	Saito T	2021	Case report (N=1); Impella-related

RecordID	First Author	Year	Exclusion Reason
			thrombus complication
1872	Chatzis G	2021	Cardiogenic shock; not cardiac arrest
1902	Le Guennec L	2020	Complication case series; not primary ECPR study
1922	Champion S	2021	Case report (N=1); non-coronary etiology (TTP)
1952	Ravishankar R	2020	Conference abstract only (EuroELSO 2020 poster); no full-text publication found
1971	Mazzeffi MA	2020	Editorial/commentary; not original research
2027	Montero-Cabezas JM	2019	Technical paper about closure device; not ECPR study
2033	Garan AR	2019	Cardiogenic shock comparison; not cardiac arrest
2035	Michels G	2019	Consensus statement/guideline review; not original research
2085	Nathan S	2019	Conference abstract only (~300 words); insufficient detail for phenotype classification
2087	Valluri K	2019	Case report; focused on cardiogenic shock management
2107	Taniguchi Y	2018	Cardiogenic shock;

RecordID	First Author	Year	Exclusion Reason
			not cardiac arrest
2137	Ming-Ting Chou	2018	Case report (N=1); cardiac arrest during LV lead implantation
2157	Ito K	2018	Complication case report (bilateral arm gangrene + HIT)
2160	Lalova I	2018	Case report (N=1); refractory VT alternating with PEA
2167	Floerchinger B	2018	No PCI/coronary angiography data; biomarker study (NSE)
2184	Lee JW	2017	Case report (N=1); non-coronary etiology (tachycardia-induced cardiomyopathy)
2201	Avalli L	2017	Conference abstract only (Euro-ELSO 2017); no standalone full-text publication
2218	Prondzinsky R	2017	Review article; not original research
2219	Fox H	2017	Case report (N=1); myocarditis (non-coronary etiology)
2223	Hungerford S	2017	Case report (N=1); transplant vasculopathy (not ECPR for cardiac arrest)
2240	Kruger A	2016	Conference abstract only

RecordID	First Author	Year	Exclusion Reason
			(ACCA 2016); no PCI data reported; no full-text publication found
2259	Dabbouseh NM	2016	Case report (N=1)
2277	Kuroki N	2015	Conference abstract (AHA 2015); duplicate of RecordID 657 (Kuroki N 2017 full-text publication)
2284	Soeholm H	2015	Not ECPR population; refractory OHCA managed without ECPR (conservative)
2331	Khaladj N	2013	Mixed cardiogenic shock population; not specifically cardiac arrest/ECPR

Summary of Exclusion Reasons:

Reason	Count
Case report / series <5 patients	26
No PCI/coronary angiography data reported	14
Not ECPR population (cardiogenic shock, post-arrest shock, not cardiac arrest)	13
Not original research (review/editorial/letter/guideline/commentary/simulation)	13
Conference abstract only (insufficient data)	5
Non-English / PDF unavailable	4
Duplicate	3

Reason	Count
Pediatric population	3
Cannot classify ROSC-at-PCI phenotype	2
Other (animal study, geospatial modelling)	2

Note: Some studies had multiple exclusion reasons; the primary reason is listed.

Table S4. Phenotype Classification Evidence for Included Studies

Systematic Review: PCI Without Sustained ROSC Under ECPR — A Phenotype-Oriented Descriptive Systematic Review

Each included study was classified as DEFINITE or PROBABLE based on whether the study explicitly documented that patients had no sustained ROSC at the time of PCI initiation. Classification followed the workflow: Claude draft with evidence quote and confidence level, followed by Lead (human) review and confirmation.

Classification Definitions

Classification	Definition
**DEFINITE**	Study explicitly states "no sustained ROSC" or equivalent; or explicitly excludes patients who achieved sustained ROSC before intervention
**PROBABLE**	Clinical workflow strongly implies no sustained ROSC at PCI (e.g., refractory arrest -> ECPR -> immediate catheterization), but no explicit ROSC-at-PCI statement

Confidence Levels

Level	Description
**HIGH**	Direct quote clearly supports classification; no ambiguity
**MEDIUM**	Reasonable inference from study design/workflow; some ambiguity in ROSC timing
**LOW**	Classification based on indirect

Level	Description
	evidence; multiple interpretations possible

Included Studies (N=27)

#	Study	Year	Classification	Confidence	Evidence Quote	Evidence Location
1	Alhuneafat L	2025	PROBABLE	MEDIUM	"ELSO Registry ECPR cohort (ECMO during CPR); 576 OHCA patients; 138 (24.3%) received PCI identified by CPT codes. ELSO ECPR definition implies no sustained ROSC at cannulation but no explicit ROSC-at-PCI statement."	Methods
2	Righetti S	2025	PROBABLE	MEDIUM	"Patients who fail to achieve	Methods

#	Study	Year	Classification	Confidence	Evidence Quote	Evidence Location
					sustained ROSC after 10-15 min...classified as having refractory OHCA; 122 (94.6%) underwent immediate CAG after ECMO initiation before ICU admission.”	
3	Nakashima T	2025	DEFINITE	HIGH	“251 (37%) patients had refractory cardiac arrest despite ECPR initiation and subsequently underwent primary PCI”	Abstract
4	Takeuchi T	2024	PROBABLE	MEDIUM	“ECPR cohort with PCI;	Full-text

#	Study	Year	Classification	Confidence	Evidence Quote	Evidence Location
					workflow suggests no sustained ROSC”	
5	Crespo-Diaz R	2024	DEFINITE	HIGH	“Patients with sustained ROSC (>20 minutes) were excluded”	Methods
6	Fu HY	2024	PROBABLE	MEDIUM	“ELSO definition of ECPR cohort implies no sustained ROSC”	Full-text
7	Nishimura T	2024	DEFINITE	HIGH	“Exclusion: patients who had sustained return of spontaneous circulation (ROSC) when ECMO was initiated. PCI performed in 48.6-78.3% of patients	Methods

#	Study	Year	Classification	Confidence	Evidence Quote	Evidence Location
					after ECMO.”	
8	Kim Y	2023	PROBABLE	MEDIUM	“ECPR = application of ECMO on patients who have failed to achieve sustained spontaneous circulation recovery (ROSC). Flow chart excludes ECMO applied after sustained ROSC (n=1116). ECPR defined as ECMO pump-on during cardiac massage.”	Methods + Fig 1
9	Nakashima T	2023	DEFINITE	HIGH	“SAVE-J II registry: ECPR for OHCA secondary to AMI. Transient	Methods + Results

#	Study	Year	Classification	Confidence	Evidence Quote	Evidence Location
					ROSC occurred in 24% before PCI; 76% without any ROSC before PCI.”	
10	Tonna JE	2022	PROBABLE	MEDIUM	“ELSO Registry ECPR cohort 2008-2019; 7488 adults (29% survival). Coronary angiography performed as post-cannulation management. ECPR = ECMO during CPR per ELSO definition.”	Methods + Table 2
11	Kawakami S	2022	DEFINITE	HIGH	“Inclusion: ROSC not achieved after hospital arrival.	Methods + Discussion

#	Study	Year	Classification	Confidence	Evidence Quote	Evidence Location
					Exclusion: ROSC present at hospital arrival; ROSC achieved between hospital arrival and ECPR initiation. ECPR stringently defined as VA-ECMO use in patients who did not achieve ROSC between hospital arrival and VA-ECMO initiation."	
12	Chico-Carballas JI	2021	PROBABLE	MEDIUM	"7 refractory IHCA patients underwent ECPR. ECPR = VA-ECMO in refractory cardiac	Methods + Results

#	Study	Year	Classification	Confidence	Evidence Quote	Evidence Location
					arrest to maintain perfusion of vital organs. 5/7 (71.4%) underwent coronary angiography after commencement of ECMO; 3/7 underwent PCI.”	
13	Hryniewicz K	2021	DEFINITE	HIGH	“Refractory arrest defined as sustained cardiac arrest without ROSC despite usual AHA ACLS care (per ELSO definition). 26 patients underwent ECPR; ECMO initiated in CCL.”	Methods
14	Porto I	2021	PROBABL	MEDIUM	“RCA	Methods

#	Study	Year	Classification	Confidence	Evidence Quote	Evidence Location
			E		defined by the lack of return to spontaneous circulation (ROSC) after 10 min of standard advanced CPR. 29 patients underwent ECLS for refractory CA or cardiogenic shock complicating ACS.”	
15	Radsel P	2021	PROBABLE	MEDIUM	“Refractory cardiac arrest was defined as a lack of spontaneous circulation or arterial pulsation while advanced cardiac life support measures	Methods

#	Study	Year	Classification	Confidence	Evidence Quote	Evidence Location
					are ongoing; 24 patients received PCI on ECMO”	
16	Bartos JA	2020	DEFINITE	HIGH	“Entry criteria: VF/VT OHCA with no ROSC following 3 shocks. ECMO cannulation in ED then transfer to CCL for angiography/PCI. Patients achieving hemodynamically stable ROSC during transport were treated per standard practice (diverted from ECPR protocol).”	Methods + Results

#	Study	Year	Classification	Confidence	Evidence Quote	Evidence Location
17	Boudoulas KD	2020	DEFINITE	HIGH	“ECPR for out-of-hospital refractory VT/VF. Inclusion: VT/VF refractory to 3 consecutive defibrillations (no ROSC). ECMO placed in CCL then immediate catheterization. If patient achieved ROSC they were transported to ICU (not ECPR protocol).”	Methods + Results
18	Duerschmied D	2020	DEFINITE	HIGH	“ECPR offered to patients without ROSC. ECPR defined as VA-ECMO implantation during	Methods + Discussion

#	Study	Year	Classification	Confidence	Evidence Quote	Evidence Location
					continuous CPR without ROSC or within first 20 min after ROSC with uncontrollable hemodynamic instability. CA patients without ROSC scheduled for ECPR should be transferred into CCL where VA-ECMO implanted first then coronary angiography immediately.”	
19	Lamhaut L	2018	PROBABLE	HIGH	“74 refractory OHCA patients treated with	Methods + Conclusion

#	Study	Year	Classification	Confidence	Evidence Quote	Evidence Location
					ECPR implanted by prehospital MoICU. Immediate CAG followed when indicated by PCI seems warranted in refractory CA after implantation of ECPR.”	
20	Cesana F	2018	DEFINITE	HIGH	“Refractory CA patients in whom ROSC could not be achieved after 15 min of c-CPR. ECMO team activation for refractory CA. e-CPR as bridge to coronary reperfusion in	Methods

#	Study	Year	Classification	Confidence	Evidence Quote	Evidence Location
					angiography laboratory. 63/106 refractory CA patients (59%) had coronary angiography.”	
21	Haas NL	2017	PROBABLE	MEDIUM	“ELSO Registry ECPR for refractory OHCA; 217 OHCA cases 2010-2016. ECPR = addition of VA-ECMO to patients failing conventional resuscitation. 26.3% underwent PCI.”	Methods + Results
22	Kuroki N	2017	DEFINITE	HIGH	“119 consecutive ACS patients with failure of ROSC	Methods + Results

#	Study	Year	Classification	Confidence	Evidence Quote	Evidence Location
					despite ACLS who underwent ECPR. PCI was performed in all patients (100%). Emergency CAG and PCI performed immediately after ECPR.”	
23	Dennis M	2017	DEFINITE	HIGH	“Patients with refractory cardiac arrest without return of spontaneous circulation (ROSC) were eligible for ECPR. Patients who received ECMO support for cardiogenic shock following	Methods

#	Study	Year	Classification	Confidence	Evidence Quote	Evidence Location
					ROSC were excluded. Angiography in 54%; PCI in 27%.”	
24	Stub D	2015	DEFINITE	HIGH	“CHEER trial: refractory OHCA treated with mechanical CPR + hypothermia + ECMO + early reperfusion. Patients with cardiac arrest and ROSC followed by cardiogenic shock who were later treated with VA-ECMO are not eligible. PCI in 11/26 (42%).”	Methods + Results

#	Study	Year	Classification	Confidence	Evidence Quote	Evidence Location
25	Sakai T	2014	PROBABLE	MEDIUM	“35 shock-resistant VF patients (sustained VF at hospital arrival = no prehospital ROSC). ELS in 54% (19/35); PCI in 40% (14/35).”	Results
26	Sakamoto S	2012	PROBABLE	MEDIUM	“98 ACS patients with cardiogenic shock or cardiac arrest refractory to conventional treatment. 36/98 (36.7%) had cardiac arrest on arrival. ECLS for circulatory collapse refractory to	Methods + Results

#	Study	Year	Classification	Confidence	Evidence Quote	Evidence Location
					conventional treatment. Emergency PCI in 92/98 (93.9%).”	
27	Chen JS	2006	PROBABLE	MEDIUM	“36 AMI patients with refractory shock/arrest requiring ECLS rescue. 30/36 (83.3%) received ECLS during CPR because spontaneous circulation failed to return. 7 patients underwent angioplasty; 28 underwent CABG.”	Methods + Results

---

Classification Summary

Classification	Count	Percentage

Classification	Count	Percentage
DEFINITE	13	48.1%
PROBABLE	14	51.9%
**Total**	**27**	**100%**

Confidence	Count	Percentage
HIGH	15	55.6%
MEDIUM	12	44.4%
LOW	0	0%
**Total**	**27**	**100%**

Notes

1. All classifications were confirmed by the Lead investigator (human reviewer).
2. DEFINITE classifications required explicit statements about ROSC exclusion or explicit documentation that patients had no sustained ROSC at the time of coronary intervention.
3. PROBABLE classifications were assigned when the ECPR workflow strongly implied no sustained ROSC at PCI (e.g., ELSO registry ECPR definition, refractory arrest protocol leading directly to catheterization) but lacked an explicit statement about ROSC status at the specific moment of PCI initiation.
4. No studies were classified as UNCERTAIN among the 27 included studies. Studies with uncertain phenotype classification were excluded from the main analysis and are documented separately.
5. Electrical activity (PEA, VT, VF, organized rhythm) without mechanical circulation was not counted as ROSC per protocol definitions.
6. Intermittent ROSC was permitted and did not affect classification, provided sustained ROSC (≥20 minutes per Utstein convention) was not achieved before PCI.

Table S5. PRISMA 2020 Checklist

Systematic Review: PCI Without Sustained ROSC Under ECPR — A Phenotype-Oriented Descriptive Systematic Review

Section/Topic	Item #	Checklist Item	Location
**TITLE**			

Section/Topic	Item #	Checklist Item	Location
Title	1	Identify the report as a systematic review	Title
**ABSTRACT**			
Abstract	2	See the PRISMA 2020 for Abstracts checklist	Abstract
**INTRODUCTION**			
Rationale	3	Describe the rationale for the review in the context of existing knowledge	Introduction ¶1–2
Objectives	4	Provide an explicit statement of the objective(s) or question(s) the review addresses	Introduction ¶3
**METHODS**			
Eligibility criteria	5	Specify the inclusion and exclusion criteria for the review and how studies were grouped for the syntheses	Methods §3
Information sources	6	Specify all databases, registers, websites, organisations, reference lists and other sources searched or consulted to identify studies	Methods §2
Search strategy	7	Present the full search strategies for all databases, registers and	Table S1

Section/Topic	Item #	Checklist Item	Location
		websites, including any filters and limits used	
Selection process	8	Specify the methods used to decide whether a study met the inclusion criteria of the review, including how many reviewers screened each record and each report retrieved, whether they worked independently, and if applicable, details of automation tools used in the process	Methods §3
Data collection process	9	Specify the methods used to collect data from reports, including how many reviewers collected data from each report, whether they worked independently, any processes for obtaining or confirming data from study investigators, and if applicable, details of automation tools	Methods §4

Section/Topic	Item #	Checklist Item	Location
		used in the process	
Data items	10a	List and define all outcomes for which data were sought. Specify whether all results that were compatible with each outcome domain in each study were sought, and if not, the methods used to decide which results to collect	Methods §4
	10b	List and define all other variables for which data were sought. Describe any assumptions made about any missing or unclear information	Methods §4; Assumptions log
Study risk of bias assessment	11	Specify the methods used to assess risk of bias in the included studies, including details of the tool(s) used, how many reviewers assessed each study and whether they worked independently, and if applicable, details of automation tools used in the process	Methods §5

Section/Topic	Item #	Checklist Item	Location
Effect measures	12	Specify for each outcome the effect measure(s) used in the synthesis or presentation of results	N/A (descriptive synthesis)
Synthesis methods	13a	Describe the processes used to decide which studies were eligible for each synthesis	Methods §6
	13b	Describe any methods required to prepare the data for presentation or synthesis, such as handling of missing summary statistics, or data conversions	Methods §6
	13c	Describe any methods used to tabulate or visually display results of individual studies and syntheses	Methods §6
	13d	Describe any methods used to synthesize results and provide a rationale for the choice(s). If meta-analysis was performed, describe the model(s), method(s) to identify the presence and extent of statistical	Methods §6 (no meta-analysis)

Section/Topic	Item #	Checklist Item	Location
		heterogeneity, and software package(s) used	
	13e	Describe any methods used to explore possible causes of heterogeneity among study results	Methods §6 (sensitivity analysis)
	13f	Describe any sensitivity analyses conducted to assess robustness of the synthesized results	Methods §6 (DEFINITE-only)
Reporting bias assessment	14	Describe any methods used to assess risk of bias due to missing results in a synthesis	N/A (no meta-analysis)
Certainty assessment	15	Describe any methods used to assess certainty (or confidence) in the body of evidence for an outcome	N/A (descriptive synthesis)
**RESULTS**			
Study selection	16a	Describe the results of the search and selection process, from the number of records identified in the search to the number of studies included in the review, ideally	Results §1; Figure 1

Section/Topic	Item #	Checklist Item	Location
		using a flow diagram	
	16b	Cite studies that might appear to meet the inclusion criteria, but which were excluded, and explain why they were excluded	Table S3
Study characteristics	17	Cite each included study and present its characteristics	Results §2; Table 1
Risk of bias in studies	18	Present assessments of risk of bias for each included study	Results §6; Table S2; Figure 3
Results of individual studies	19	For all outcomes, present, for each study: (a) summary statistics for each group and (b) an effect estimate and its precision	Tables 1–3
Results of syntheses	20a	For each synthesis, briefly summarise the characteristics and risk of bias among contributing studies	Results §3–5
	20b	Present results of all statistical syntheses conducted. If meta-analysis was done, present for each the summary estimate and its	N/A (descriptive synthesis)

Section/Topic	Item #	Checklist Item	Location
		precision and measures of statistical heterogeneity. If comparing groups, describe the direction of the effect	
	20c	Present results of all investigations of possible causes of heterogeneity among study results	Results §5 (sensitivity analysis)
	20d	Present results of all sensitivity analyses conducted to assess the robustness of the synthesized results	Results §5 (sensitivity analysis)
Reporting biases	21	Present assessments of risk of bias due to missing results for each synthesis assessed	N/A (no meta-analysis)
Certainty of evidence	22	Present assessments of certainty (or confidence) in the body of evidence for each outcome assessed	N/A (descriptive synthesis)
**DISCUSSION**			
Discussion	23a	Provide a general interpretation of the results in the context of other evidence	Discussion §2
	23b	Discuss any	Discussion §4

Section/Topic	Item #	Checklist Item	Location
		limitations of the evidence included in the review	
	23c	Discuss any limitations of the review processes used	Discussion §4
	23d	Discuss implications of the results for practice, policy, and future research	Discussion §3, §5
**OTHER INFORMATION**			
Registration and protocol	24a	Provide registration information for the review, including register name and registration number, or state that the review was not registered	Methods §1 (PROSPERO CRD420251252255)
	24b	Indicate where the review protocol can be accessed, or state that a protocol was not prepared	Methods §1
	24c	Describe and explain any amendments to information provided at registration or in the protocol	Methods §7
Support	25	Describe sources of financial or non-financial support for the review, and	Funding statement

Section/Topic	Item #	Checklist Item	Location
		the role of the funders or sponsors in the review	
Competing interests	26	Declare any competing interests of review authors	Conflict of interest statement
Availability of data, code, and other materials	27	Report which of the following are publicly available and where they can be found: template data collection forms; data extracted from included studies; data used for all analyses; analytic code; any other materials used in the review	Data availability statement

From: Page MJ, McKenzie JE, Bossuyt PM, et al. The PRISMA 2020 statement: an updated guideline for reporting systematic reviews. BMJ 2021;372:n71.

Table S6. Sensitivity Analyses

Purpose: To assess robustness of descriptive findings to (a) phenotype-classification uncertainty (PROBABLE cohort) and (b) inter-registry overlap.

Cohort Sizes

Subset	N studies	N patients
Primary (DEFINITE)	13	3,320
Supportive (PROBABLE)	14	9,562
Combined (all 27)	27	12,882
**Exclude-overlap**	19	2,741

Key Outcome Comparison

Variable	DEFINITE Primary	Combined (27)	Exclude-Overlap
Survival to discharge, median [IQR]	30.3 [26.5–40.8] (n=11)	29.2 [23.1–34.2] (n=20)	31.1 [27.2–37.0] (n=12)
Survival to discharge, range	21.0–69.0	13.8–69.0	13.8–69.0
Favorable neuro CPC 1–2, median [IQR]	33.5 [16.8–45.8] (n=12)	30.3 [16.8–41.1] (n=20)	30.6 [26.8–37.8] (n=15)
Favorable neuro, range	10.4–92.0	9.8–92.0	9.8–65.0
PCI rate, range	24.0–100.0 (n=10)	24.0–100.0 (n=23)	24.0–100.0 (n=18)
TIMI 3 flow, range	69.7–84.0 (n=3)	62.4–84.0 (n=5)	62.4–84.0 (n=3)
Shockable rhythm, range	33.0–100.0 (n=13)	23.5–100.0 (n=22)	23.5–100.0 (n=16)

Interpretation

Central tendency and ranges are concordant across the three analytic subsets. The inclusion of PROBABLE studies (phenotype-inferred) did not materially shift the descriptive envelope from the DEFINITE primary cohort, and the exclude-overlap subset — in which inter-registry double counting is eliminated — produced outcomes within overlapping IQRs of both the DEFINITE and combined sets. Consistency across the three subsets supports that the observed envelope is not driven by classification uncertainty or by patient-level overlap between ELSO, SAVE-J II, and their secondary analyses.

Table S7. ROBINS-I Risk-of-Bias Assessment — 13 DEFINITE Studies

Tool: ROBINS-I (Risk Of Bias In Non-randomized Studies of Interventions) [40] **Scope:** Primary analysis cohort (13 DEFINITE studies) **Assessor:** Single reviewer (Y.H.C.); limitation noted in Methods

Domain-Level Judgements

Study	D1 Conf	D2 Sel	D3 Class	D4 Dev	D5 Miss	D6 Out	D7 Rep	**Overall**

Study	D1 Conf	D2 Sel	D3 Class	D4 Dev	D5 Miss	D6 Out	D7 Rep	**Overall**
Nakashima 2025	Mod	Mod	Low	Mod	Low	Low	Mod	**Moderate**
Crespo-Diaz 2024	Mod	Mod	Low	Mod	Mod	Low	Mod	**Moderate**
Nishimura 2024	Mod	**Ser**	Low	Mod	Mod	Low	Mod	**Serious**
Nakashima 2023	Mod	Mod	Low	Mod	**Ser**	Low	**Ser**	**Serious**
Kawakami 2022	Mod	Mod	Low	Mod	Low	Low	Low	**Moderate**
Hryniewicz 2021	**Ser**	**Ser**	Mod	Mod	Mod	Low	Mod	**Serious**
Bartos 2020	Mod	**Ser**	Low	Mod	Low	Low	Low	**Serious**
Boudoulas 2020	**Ser**	**Ser**	Low	**Ser**	Mod	Low	Mod	**Serious**
Duerschmied 2020	Mod	**Ser**	Mod	Mod	Mod	**Ser**	Mod	**Serious**
Cesana 2018	**Ser**	**Ser**	Mod	Mod	**Ser**	Low	Mod	**Serious**
Dennis 2017	Mod	**Ser**	Low	Mod	Mod	Low	Mod	**Serious**
Kuroki 2017	Mod	Mod	Low	Mod	Mod	Low	Mod	**Moderate**
Stub 2015	Mod	**Ser**	Low	Mod	Low	Low	Mod	**Serious**

Abbreviations: D1 Confounding; D2 Selection of participants; D3 Classification of interventions; D4 Deviations from intended interventions; D5 Missing data; D6 Outcome measurement; D7 Selection of reported results. Mod = Moderate; Ser = Serious.

Distribution

Overall rating	n	%
Low	0	0.0
Moderate	4	30.8
**Serious**	9	69.2
Critical	0	0.0

Comparison with JBI Appraisal

The JBI Critical Appraisal Checklists applied to the same 13 studies had classified 12 as “low concern” and 1 as “moderate.” The ROBINS-I reassessment reclassifies 9 of those 12 as Serious risk of bias. This realignment reflects ROBINS-I’s explicit probing of confounding by indication and selection bias — the dominant methodological risks in observational studies of an acutely-selected intervention — which the JBI reporting-quality framework does not probe as systematically.

Summary Rationale (per-study)

One- to two-sentence rationale behind each overall judgement, citing the specific features that drove the worst-domain score:

Nakashima (2025) [Moderate]: Large multicenter SAVE-J II registry with explicit ROSC definition (60 s palpable pulsations) and mixed-effects regression, but confounding by indication for PCI selection is intrinsic to the observational design; 6 of 36 institutions excluded for incomplete catheterization reporting raises minor selection concern.

Crespo-Diaz (2024) [Moderate]: Single-center consecutive enrollment at University of Minnesota with explicit sustained-ROSC exclusion (>20 min), but VF/VT-only inclusion limits generalizability, outcomes framed by rhythm-conversion stage rather than PCI exposure, and no survival-to-discharge endpoint explicitly reported.

Nishimura (2024) [Serious]: SAVE-J II secondary analysis restricted to the ACS subgroup with 80 patients excluded for missing data (no imputation), IABP exposure left to physician discretion (strong selection bias; IABP-PCI rate 86 percent vs. 49 percent in non-IABP), and Impella recipients excluded — residual confounding for the IABP-stratified outcomes is likely.

Nakashima (2023) [Serious]: Brief letter-to-editor format (3 pages) with 47/671 (7 percent) missing post-PCI TIMI data and no imputation strategy; denominators shift across analyses (609/618/620/623/624); cannot separate no-ROSC from transient-ROSC subgroups despite that being the target phenotype, and the Letter format limits methodological transparency.

Kawakami (2022) [Moderate]: Large JAAM-OHCA multicenter registry with the most stringent ROSC exclusion in the review (no ROSC between hospital arrival and ECMO initiation), complete 30-day CPC data, and stepwise multivariable logistic regression — residual confounding by physician selection for PCI (OR 2.30 for PCI) cannot be eliminated by design.

Hryniewicz (2021) [Serious]: Highly selected single-center cohort (n = 26) with strict entry criteria (age 18–75, bystander CPR <5 min, CPR <60 min) and 31 percent CCL-witnessed arrests inflating survival (88 percent CCL vs. 71 percent OHCA vs. 55 percent non-CCL IHCA); revascularization data pooled (PCI+CABG); generalizability severely limited.

Bartos (2020) [Serious]: Prospective MMRC consortium description with a good protocol and complete follow-up, but the N = 58 denominator includes 13 patients who died before ECMO was attempted (inflating apparent mortality of the actual ECPR-PCI cohort); 4-month enrollment window, VF/VT-only inclusion, and program-description framing (not a hypothesis-driven cohort).

Boudoulas (2020) [Serious]: Very small single-center series (N = 16, 6 PCI) with inclusion criteria changed twice mid-study (March 2018 and October 2018 broadened witnessed/bystander/lactate requirements); one patient counted as two events; complications combined into pooled categories that cannot be disaggregated; statistical power inadequate for any inference.

Duerschmied (2020) [Serious]: Mixed OHCA/IHCA (56 percent IHCA) single-center registry; no CPC or neurological outcome data reported at all — only in-hospital survival, substantially limiting outcome measurement for a cardiac-arrest study; the ECPR definition permits an unstable-early-ROSC subset that may dilute the no-ROSC phenotype; 21/273 (7.7 percent) excluded for missing data.

Cesana (2018) [Serious]: Propensity-score matching explicitly failed to achieve covariate balance (authors acknowledge in the Discussion); exact PCI-within-ECPR count not reported despite being the study's subject; Table 3 dispersion statistics ambiguously labeled; ischemia-only selection (critical coronary plaque required) creates survivorship-style spectrum bias.

Dennis (2017) [Serious]: Two-center Australian case series (N = 37) with 68 percent IHCA and angiography/PCI performed at clinician discretion without a standardized pathway; the PCI subgroup (N = 9) is too small for any stratified outcome analysis; abstract and Table 2 shockable-rhythm counts differ by one patient, suggesting minor classification inconsistency.

Kuroki (2017) [Moderate]: Single-center consecutive enrollment with a gold-standard phenotype (all 119 had refractory arrest with immediate ECPR+PCI, 100 percent PCI rate) and rich timing data; the 11-year study period (2005–2016)

introduces practice-evolution confounding; 69 percent IHCA limits OHCA generalizability; 16 patients excluded pre-enrollment for no CAG/PCI.

Stub (2015) [Serious]: CHEER pilot (N = 26) with prospective registration (NCT01186614) and explicit post-ROSC-cardiogenic-shock exclusion, but IHCA inclusion was “at the discretion of the attending critical care physician” (explicit selection bias) and OHCA required VF with bystander CPR within 10 min, producing a highly favorable selected population (54 percent survival; 100 percent CPC 1 among survivors is implausibly high for the general ECPR population).

Table S8. Complete Extraction Dataset (27 Studies × 93 Variables)

The complete structured extraction dataset for all 27 included studies (93 variables spanning study identification, selection profile [Axis 1], procedural feasibility [Axis 2], outcomes [Axis 3], and risk-of-bias fields) is provided as a separate Microsoft Excel file, **“Table S8 — Complete Extraction Dataset.xlsx”**, accompanying this Supplementary Materials document.

Supplementary Figures

Figure S1. JBI Critical Appraisal summary: distribution of overall reporting-quality concern across all 27 included studies (Low / Moderate / High concern).

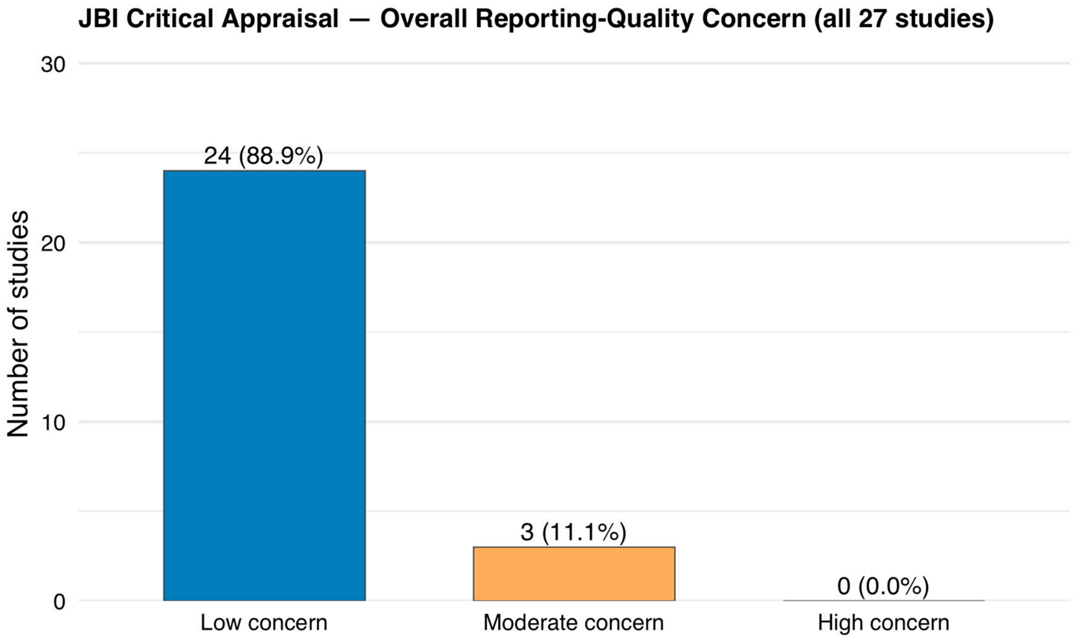

Figure S2. Exploratory relationship between shockable-rhythm proportion and survival to hospital discharge across reporting studies. Point size proportional to sample size; colour indicates phenotype classification.

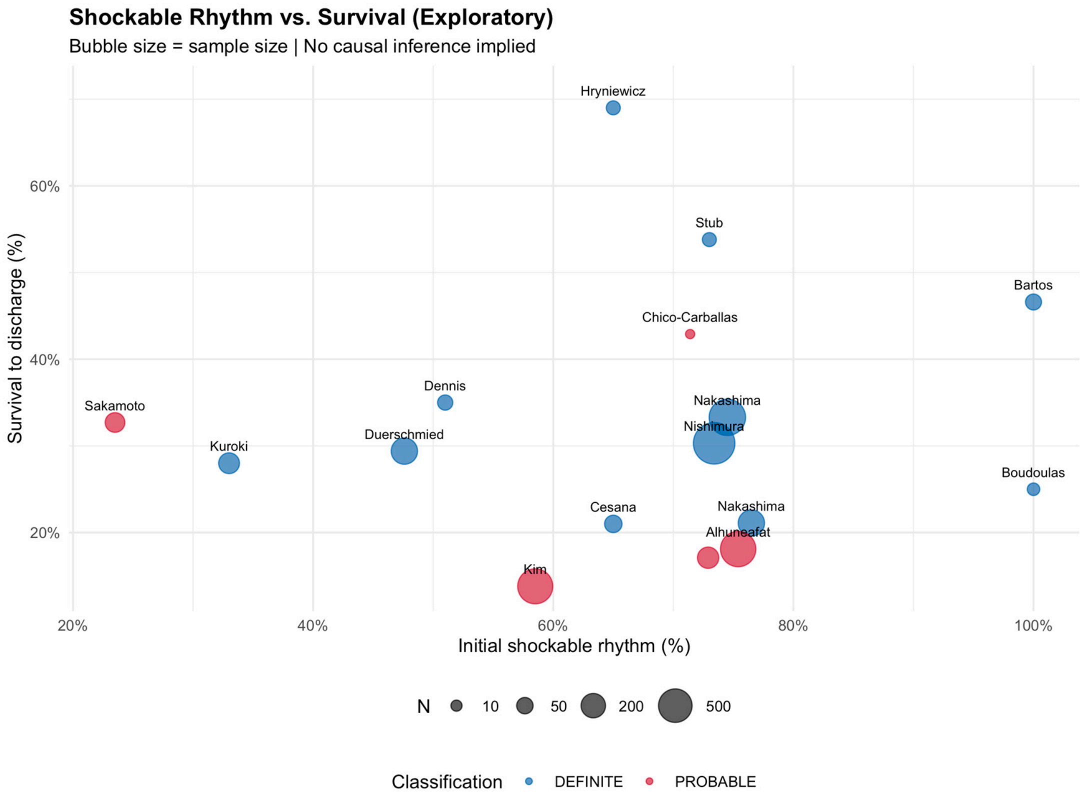

Figure S3. Combined outcome panel (exploratory): survival to discharge, favourable neurological outcome (CPC 1–2), and ECMO weaning across reporting studies.

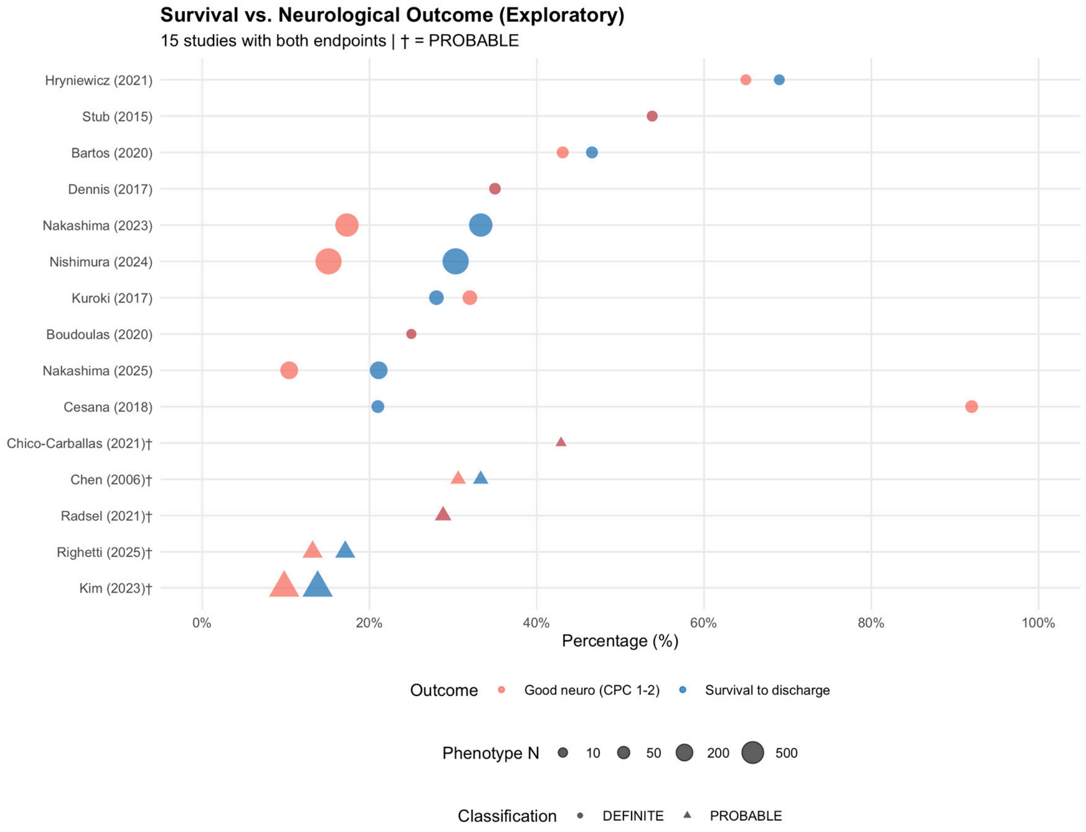
Survival vs. Neurological Outcome (Exploratory)
15 studies with both endpoints | † = PROBABLE
Hryniewicz (2021)
Stub (2015)
Bartos (2020)
Dennis (2017)
Nakashima (2023)
Nishimura (2024)
Kuroki (2017)
Boudoulas (2020)
Nakashima (2025)
Cesana (2018)
Chico-Carballas (2021)†
Chen (2006)†
Radsel (2021)†
Righetti (2025)†
Kim (2023)†
0%
20%
40%
60%
80%
100%
Percentage (%)
Outcome
Good neuro (CPC 1-2)
Survival to discharge
Phenotype N
10
50
200
500
Classification
DEFINITE
PROBABLE
